# Chronological and genetic analysis of an Upper Palaeolithic female infant burial from Borsuka Cave, Poland

**DOI:** 10.1016/j.isci.2023.108283

**Published:** 2023-10-23

**Authors:** Helen Fewlass, Elena I. Zavala, Yoann Fagault, Thibaut Tuna, Edouard Bard, Jean-Jacques Hublin, Mateja Hajdinjak, Jarosław Wilczyński

**Affiliations:** 1 Department of Human Evolution, Max Planck Institute for Evolutionary Anthropology, 04103 Leipzig, Germany; 2 Ancient Genomics Lab, Francis Crick Institute, London NW1 1AT, UK; 3 Department of Evolutionary Genetics, Max Planck Institute for Evolutionary Anthropology, 04103 Leipzig, Germany; 4 Department of Cell and Molecular Biology, University of California, Berkeley, Berkeley, CA 94720-3200, USA; 5 Department of Biology, San Francisco State University, San Francisco, CA 94132, USA; 6 CEREGE, Aix Marseille Université, CNRS, IRD, INRA, Collège de France, Technopôle de l’Arbois BP 80, 13545 Aix-en-Provence Cedex 4, France; 7 Chaire de Paléoanthropologie, CIRB (UMR 7241 – U1050), Collège de France, 75231 Paris, France; 8 Institute of Systematics and Evolution of Animals, Polish Academy of Sciences, Sławkowska 17, 31-016 Krakow, Poland

**Keywords:** Genetics, Paleogenetics

## Abstract

Six infant human teeth and 112 animal tooth pendants from Borsuka Cave were identified as the oldest burial in Poland. However, uncertainties around the dating and the association of the teeth to the pendants have precluded their association with an Upper Palaeolithic archaeological industry. Using <67 mg per tooth, we combined dating and genetic analyses of two human teeth and six herbivore tooth pendants to address these questions. Our interdisciplinary approach yielded informative results despite limited sampling material, and high levels of degradation and contamination. Our results confirm the Palaeolithic origin of the human remains and herbivore pendants, and permit us to identify the infant as female and discuss the association of the assemblage with different Palaeolithic industries. This study exemplifies the progress that has been made toward minimally destructive methods and the benefits of integrating methods to maximize data retrieval from precious but highly degraded and contaminated prehistoric material.

## Highlights

• Interdisciplinary analyses associate Borsuka Cave burial with the Upper Palaeolithic
• Human remains and herbivore tooth pendants are directly dated to >30,000 years old
• Genetic analyses demonstrate this is the earliest known female infant burial
• Minimally invasive methods used <67 mg of tooth dentine for multiple analyses

## Graphical abstract

**Figure:**
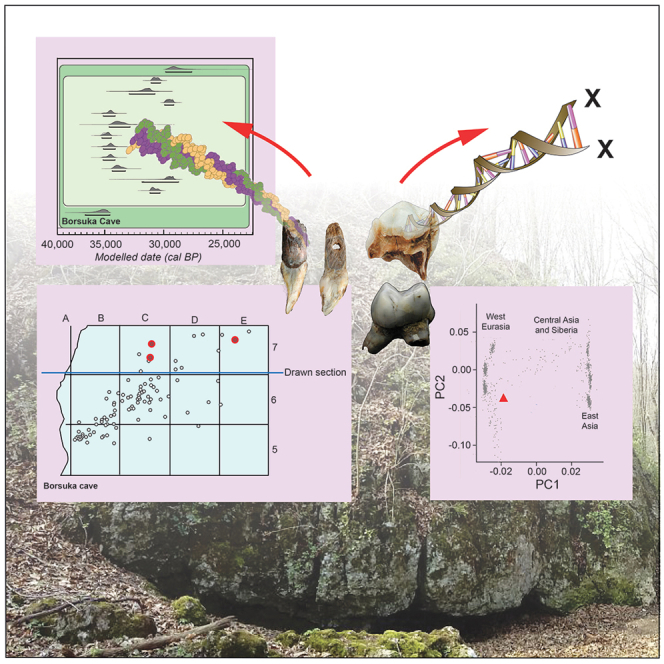

## Introduction

**Figure 1:**
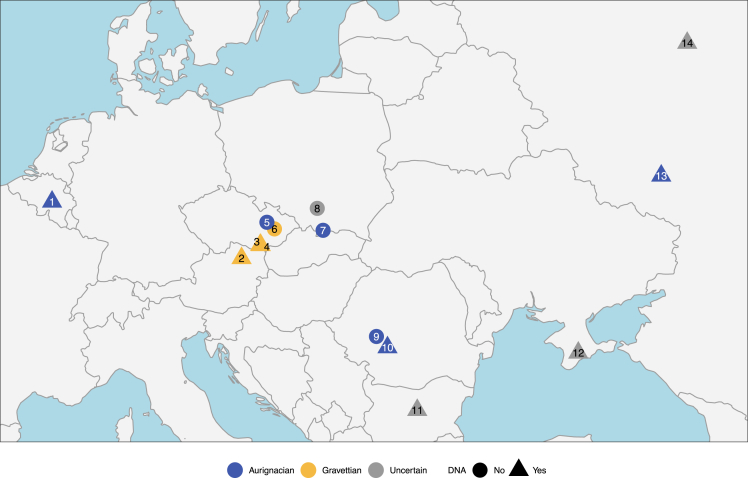
Map of Upper Palaeolithic sites mentioned in the text from Central and Eastern Europe containing human remains and the archaeological complex with which they are currently associated Sites number key: 1. Goyet Cave, 2. Krems-Wachtberg, 3. Dolní Věstonice, 4. Pavlov, 5. Mladeč, 6. Předmostí, 7. Oblazowa Cave, 8. Borsuka Cave, 9. Peștera Cioclovina, 10. Peștera Muierii, 11. Bacho Kiro Cave, 12. Buran Kaya III, 13. Kostenki, 14. Sunghir.

Human remains from the Upper Palaeolithic (UP) in Eurasia are sparse, but the period is marked by the increasing visibility of human burials in the archaeological record, typically associated with the Gravettian culture (∼34 - 24 ka cal BP) and occasionally even earlier (∼39 - 34 ka cal BP). Eastern Central Europe has a rich record of Palaeolithic archaeology with several sites containing human remains (Figure 1), which are associated with different technological assemblages. Directly dated human remains at Mladeč Cave (Czech Republic),^1^ Oblazowa Cave (Poland),^2^^,^^3^^,^^4^^,^^5^^,^^6^ Peștera Muierii^7^ and Peștera Cioclovina^8^ in Romania, and Troisième Caverne of Goyet (individuals Q116-1 and Q376-3) in Belgium^9^^,^^10^ all fall within the ∼37 - 33 ka cal BP range and are tentatively associated with Aurignacian artifacts. The Kostenki-Borshchevo complex in Russia spans different periods of the UP and has yielded numerous human remains, including the burial of Kostenki 14 (∼39 - 37 ka cal BP), which is also associated with the Aurignacian.^11^^,^^12^ Isolated human remains from Buran Kaya III in Crimea^13^ are associated with Gravettian assemblages, but this is in contrast with their early direct age of ∼36.8–35.7 ka cal BP. In Central Europe, the Gravettian is split into the Early Gravettian (∼34 - 30 ka cal BP), Pavlovian (∼31 - 29 ka cal BP) and Late Gravettian/Willendorf Kostenkian (∼29 - 24 ka cal BP).^14^^,^^15^ The Pavlovian period is characterized by burials containing red ochre and grave goods such as seen at Dolní Věstonice-Pavlov^16^^,^^17^^,^^18^^,^^19^ and Předmostí^14^^,^^20^ in Czech Republic. Whilst UP child burials have been identified in the region, including the burial of infants at Krems-Wachtberg (Austria),^21^^,^^22^^,^^23^ they are extremely rare. Much more elaborate UP burials have been found at Sunghir (Russia), containing multiple individuals, abundant ochre, and an exceptionally rich assemblage of grave goods.^24^^,^^25^ Several attempts at directly dating these individuals produced vastly differing results, likely due to issues of contamination, thus contributing to uncertainty around the cultural association of the site. The most recent and rigorous compound specific dates place the burials at Sunghir between 35.8 and 32 ka cal BP.^11^^,^^24^^,^^25^^,^^26^^,^^27^^,^^28^^,^^29^^,^^30^

**Figure 2:**
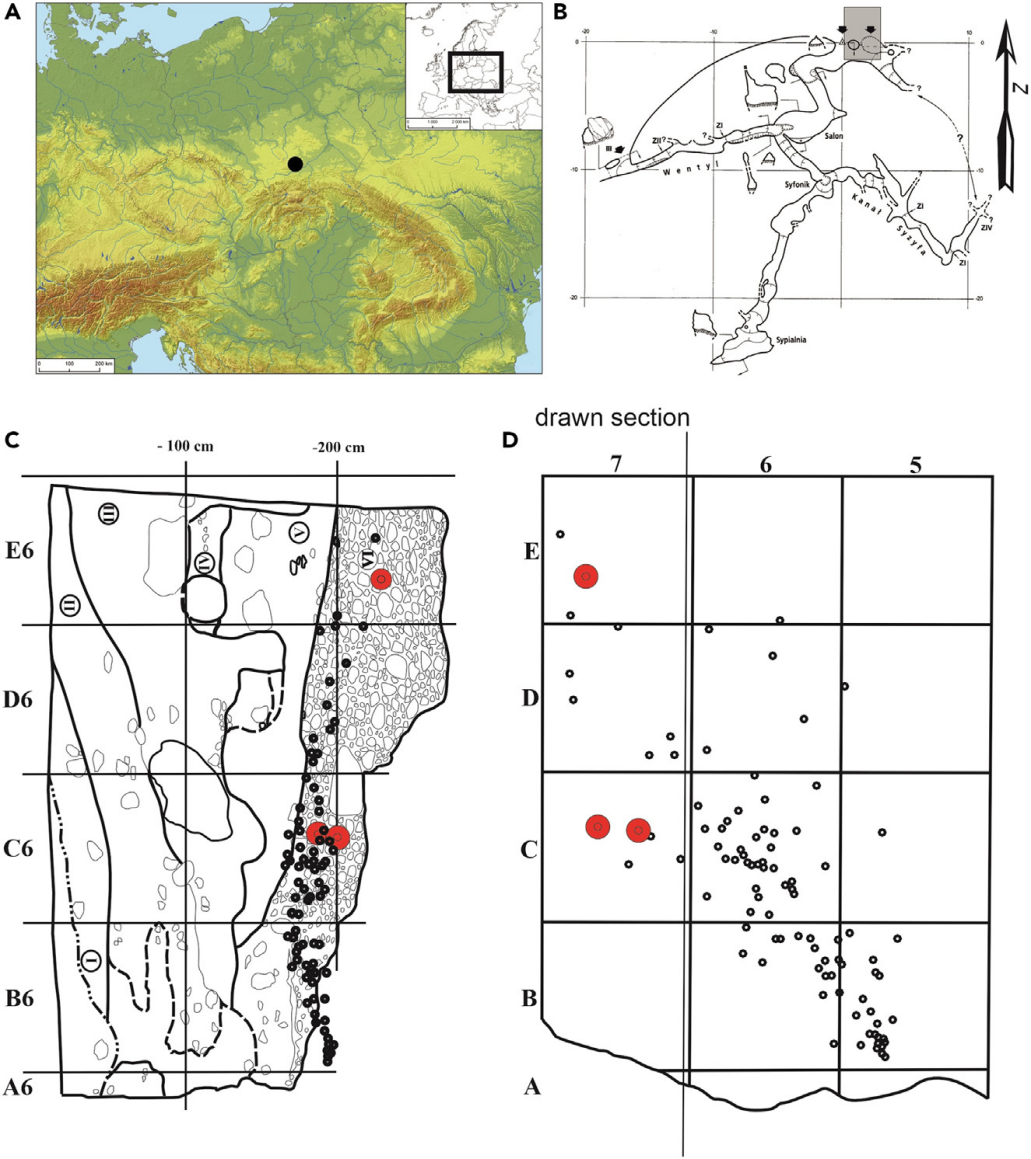
Visuals of Borsuka Cave location, site plans, and stratigraphic profile (A) Map showing the location of Borsuka Cave within Europe. (B) Plan view of Borsuka Cave showing the 2008–2010 excavation trench (gray shaded rectangle). (C) Stratigraphic profile with the location of ungulate tooth pendants (black circles) and human remains (red circles). (D) Plan view of Layer VI showing the location of human remains and pendants. Note that three human teeth found *in situ* are plotted but three were found during wet-sieving so lack exact 3D coordinates. In C and D, each square is 1 m^2^.

Borsuka Cave (50°9′53.94″N; 19°42′12.23″E), located in the Szklarka River valley in southern Poland (Figure 2A), adds important data to this discussion. Excavations next to the entrance of the cave in 2008–2010 (Figure 2B) uncovered six deciduous human teeth in association with 112 pierced teeth of Steppe wisent/aurochs (*Bison priscus*/*Bos bison*; n *= 78*) and European elk (*Alces alces*; n *= 34*), which have traces of ochre on them.^31^^,^^32^ Seven layers were excavated and separated into a lower Pleistocene unit (Layers VII - V) and an upper Holocene unit (Layers IV - I). At the base of the sequence, overlying bedrock, Layer VII was archaeologically sterile. In Layer VI (from 150 to 250 cm depth), the human remains and pierced teeth were found spread over several m^2^ in an NW linear distribution down the slope from the cave entrance. The pendants were concentrated by the southern wall of the trench at depths of 160–220 cm, with a few in the central and northern parts of the trench (Figure 2). The human teeth were located to the west of the concentration of pendants, in the northwest corner of the trench within squares C7, D7, and E7 (Figures 2C and 2D). These teeth were all identified as belonging to a child aged 12–18 months. Given the young age, it was not possible to determine the sex based on morphology, but the consistent phase of dentition indicated they likely belonged to one individual. Layer VI contained the human remains, perforated ungulate teeth, and one distal fragment of a flint blade but was otherwise archaeologically sterile. The overlying Layer V was also archaeologically and palaeontologically sterile but associated with Last Glacial Maximum (LGM) cooling. Layers IV-I contained Holocene assemblages spanning from the Mesolithic to modern (see^31^^,^^33^).

In addition to the human remains, over 2,000 faunal skeletal fragments were excavated from Layer VI. The assemblage contained an unusual collection of fauna including cold steppe-tundra species, such as *Rangifer tarandus* (reindeer), *Vulpes alopex* (Arctic fox), *Equus ferus* (wild horse) and *Coelodonta antiquitatis* (woolly rhino), alongside taxa adapted to a warmer forested environment, including *Alces alces* (elk), *Bos primigenius* (aurochs), *Martes martes* (pine marten), *Lynx lynx* (Eurasian lynx)*, Castor fiber* (beaver) and *Meles meles* (badger).^33^ The majority of the Pleistocene skeletal remains likely represent a natural accumulation, with signs of carnivore modifications on 1.7% of the mammalian and bird bones and no signs of anthropogenic modification aside from the pierced teeth.

Two of the herbivore tooth pendants were directly radiocarbon dated to 32,890 - 30,760 cal BP at 95.4% probability (Poz-32394: 27,350 ± 450 ^14^C BP; ^14^C errors reported at 1σ) and 29,900–29,120 cal BP (Poz-38236: 25,150 ± 160 ^14^C BP). A reindeer metatarsus from the same layer was dated to 31,060 - 30,270 cal BP (Poz-38237: 26,430 ± 180 ^14^C BP).^32^^,^^33^ While the dates indicated a Mid-Upper Palaeolithic age for the Layer VI assemblage, the lack of agreement between the dates from the two pendants at the 95% range suggested that the pendants are not strictly contemporaneous. This, along with the distribution of the herbivore and human teeth, leaves open the question of the association between the pendants and the human remains, necessitating further chronological investigation. However, due to their small size, direct dating of the human teeth was initially determined to be impossible.

Although alternative theories have been discussed,^32^ the absence of associated “domestic” archaeology led to the interpretation that the assemblage represents an infant burial and grave goods that were disturbed post-deposition, resulting in the absence of a burial pit and their linear spread down the slope.^32^ The lack of any Palaeolithic archaeology necessitates relying on the dating and stylistic analysis of the pendants for determining the cultural association of the burial. The existing pendant ^14^C dates are roughly contemporaneous with Pavlovian burials ∼300-200 km away at Dolní Věstonice-Pavlov and Předmostí (Czech Republic)^14^^,^^16^^,^^17^^,^^18^^,^^19^^,^^20^ and infant burials ∼400 km away at Krems-Wachtberg (Austria).^21^^,^^22^^,^^23^ However, the typology of the pendants themselves are similar to those found at Mladeč^32^ and suggest a Late Aurignacian association. In addition, there have been numerous examples where human remains assumed to be Palaeolithic in origin in fact represent Holocene intrusions into Palaeolithic contexts.^34^^,^^35^^,^^36^^,^^37^^,^^38^^,^^39^ Given the small nature of fragmented deciduous human teeth and presence of Holocene archaeology in the upper layers, the possibility of this being the case at Borsuka Cave could not be ruled out.

**Figure 3:**
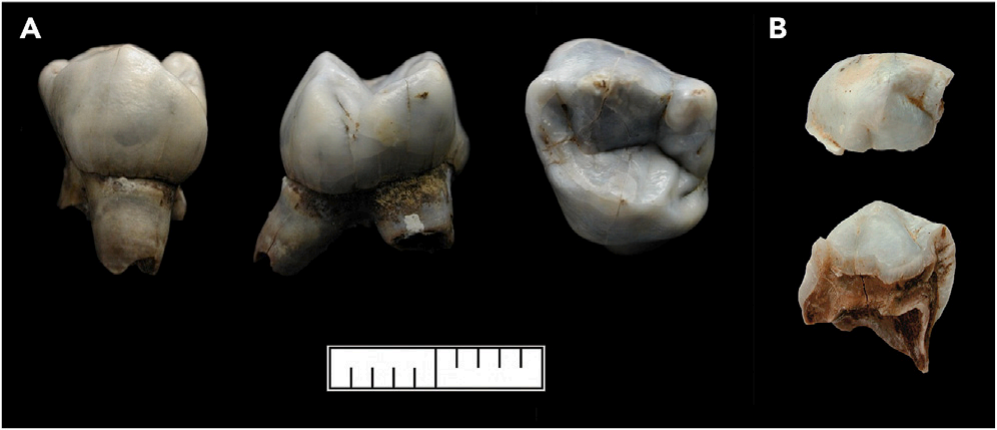
The two human teeth from Borsuka cave analyzed in the study (A) The deciduous molar C7/675 (uldm1) and (B) molar fragment C7/683. Scale bar is 1 cm.

**Figure 4:**
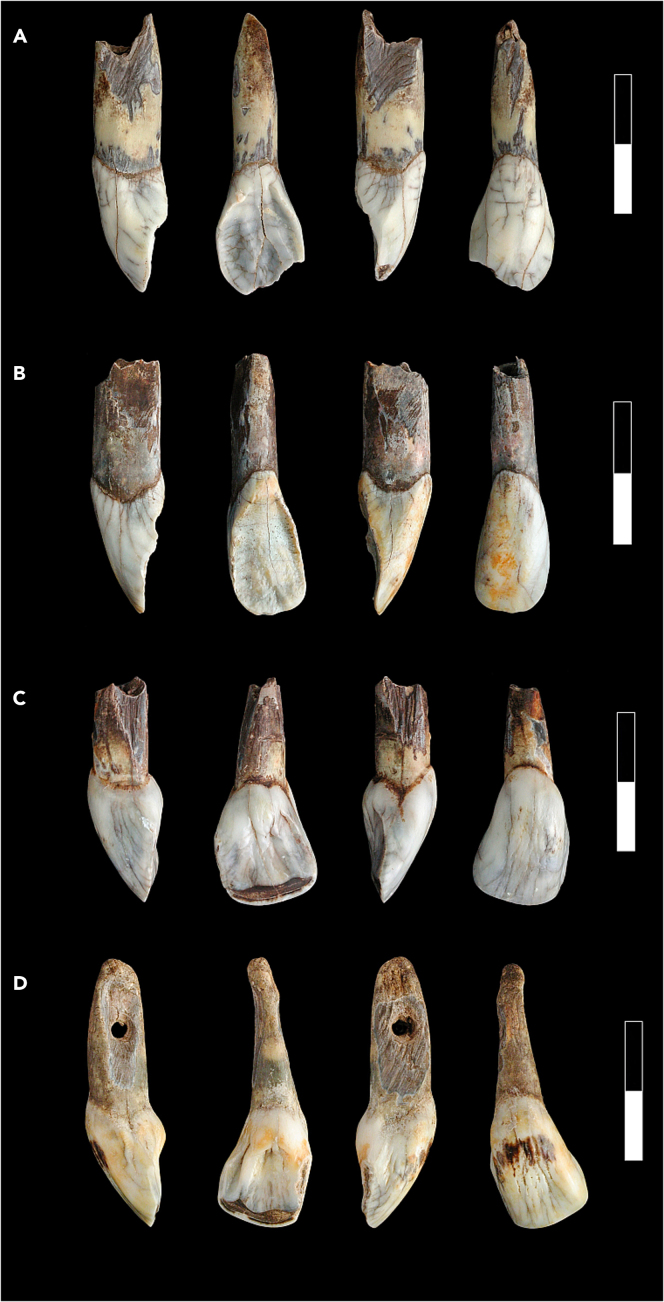
Four pendants made of large ungulate teeth from Borsuka Cave dated in the study (A) European elk (*Alces alces*) left lower incisor, sample ID: C7/656; (B) European elk (*Alces alces*) left lower incisor, sample ID: B5/910; (C) Bos/Bison (*Bos primigenius/Bos bison*) right lower incisor, sample ID: B5/913; (D) Bos/Bison (*Bos primigenius/Bos bison*), sample ID: C7/658. Scale bar is 2 cm.

Significant advances have been made in recent years for the radiocarbon dating and genetic analysis of ancient and highly degraded skeletal material, including reducing sample sizes for dating,^19^^,^^40^^,^^41^^,^^42^ combining multi-method extractions from a single sample,^43^ non-destructive pre-screening of organic preservation,^44^ treatments for removing contaminant DNA and/or enriching for endogenous ancient DNA (aDNA),^45^^,^^46^^,^^47^^,^^48^^,^^49^ and non-destructive aDNA extraction from osseous artifacts.^50^ Such developments enable biomolecular analyses of material which were previously impossible. Considering the expected age and very limited amount of material available from the human and pierced herbivore teeth from Borsuka, we devised a multi-disciplinary study to investigate (i) if the human teeth originate from a single individual, (ii) the chronological relationship of the human teeth and pendants, and (iii) the association of the assemblage to different Palaeolithic cultures. In order to confirm the Palaeolithic origin of the teeth and ascertain if the human remains are contemporaneous with the pendants, we undertook radiocarbon dating of two deciduous human teeth (Figure 3) and six of the pierced herbivore teeth (Figure 4). From the human material, we selected two teeth (C7/675, C7/683) with partially intact roots to preserve the crown morphology and selected herbivore pendants from different squares to test across the scattered assemblage. Ancient DNA analysis was attempted on the same two human teeth to determine if they belonged to one individual and to see how this individual related to other Upper Palaeolithic individuals. The assemblage at Borsuka Cave represents a challenge in terms of the extremely limited material available, and the inherent issues of poor preservation and contamination of ancient samples for both ^14^C and aDNA. Nevertheless, we aimed to maximise data retrieval from extremely small samples to provide additional information about the Borsuka assemblage within the broader context of the UP.

## Results

### Radiocarbon dating

The collagen yield of the two human teeth (4.3–8.5%) was within the range of the herbivore teeth (3.0–9.1%), consistent with degraded Palaeolithic material but all falling well above the ∼1% minimum required for dating. The elemental values (C%, N%, C/N) of all extracts fall within the commonly accepted ranges of well-preserved collagen (C%: ∼30–45%; N%: ∼11–16%; C/N: 2.9–3.6), indicating that the collagen extracts were suitable for radiocarbon dating^51^ (Table 1). However, several fall at the limit (3.5–3.6) of the accepted C/N range, indicating low levels of contamination may be present in the samples.^52^^,^^53^ For C7/675, C7/658, C6/494, sufficient collagen was available for additional quality analysis using Fourier Transform Infrared Spectroscopy (FTIR). All extracts had FTIR spectra characteristic of ancient bone collagen,^54^^,^^55^^,^^56^^,^^57^ but the higher intensity of the peak at ∼2900 cm^−1^ in the spectra of the human tooth C7/675 may be indicative of external contamination (Figure S1).

**Table 1:** Pretreatment and radiocarbon dating of humans and herbivore pendants from Borsuka Cave

Sample ID	Material	Sampled (mg)	Collagen (mg)	% Collagen	δ^13^C (‰)	δ^15^N (‰)	%C	%N	C/N	AMS lab number	combined BP	combined err	95.4% cal BP	*n*
C7/675	Human tooth - uldm1	41.1	3.5	8.5	−20.3	14.9	42.9	13.7	3.6	Aix-12047	25100	140	29860–29090	2
C7/683	Human tooth - dm	23.4	1	4.3	−20.1	13.6	39.7	13.3	3.5	Aix-12058	26610	240	31160–30320	3
C7/656	Elk incisor pendant	34.3	2.6	7.6	−20.6	4.7	42.3	14.2	3.5	Aix-12041	29310	240	34350–33260	2
C7/658	Bos/Bison incisor pendant	48.5	4.4	9.1	−20.3	7.4	42.7	15.2	3.3	Aix-12042	30100	260	35190–34150	2
C6/479	Bos/Bison incisor pendant	53.9	3.6	6.7	−19.6	6.5	41.8	15	3.3	Aix-12043	30080	260	35170–34130	2
C6/494	Elk incisor pendant	67.4	4.5	6.7	−19.8	2.3	42.7	15.1	3.3	Aix-12044	30460	190	35290–34480	3
B5/910	Elk incisor pendant	49.2	1.5	3	−20.4	3.1	37.8	13.4	3.3	Aix-12045	29310	370	34610–33860	3
B5/913	Bos/Bison incisor pendant	49.9	2.9	5.8	−20.1	6.3	40.4	14.5	3.3	Aix-12046	30080	260	35170–34140	2

Combined dates of multiple replicates (*n*) are reported here with individual replicate dates and X^2^ values reported in Table S1.

The bulk collagen δ^13^C and δ^15^N values of the two human teeth fall within the range of stable isotopic values seen for other Mid-Upper Palaeolithic humans in Eurasia,^19^^,^^23^^,^^58^^,^^59^^,^^60^^,^^61^^,^^62^ with very high δ^15^N values that are well above the herbivorous signature of the Bos/Bison and elk teeth (Figure S2). Although the δ^13^C values of the two human teeth agree within instrumental error, the δ^15^N value of C7/675 is higher than C7/683 by 1.3‰. Given that the teeth are both deciduous molars with similar formation times, the difference is more likely to be indicative of contamination, or of separate individuals, rather than palaeodietary. The elevated δ^15^N values are at the highest end of the range seen in other Mid-UP humans and in the range of two trophic levels (trophic level increase: ∼3‰–5‰) above the herbivores. This is consistent with a breastfeeding signal given the age of the infant, but without comparative adult data and further chemical analyses it is not possible to elucidate this further.

**Figure 5:**
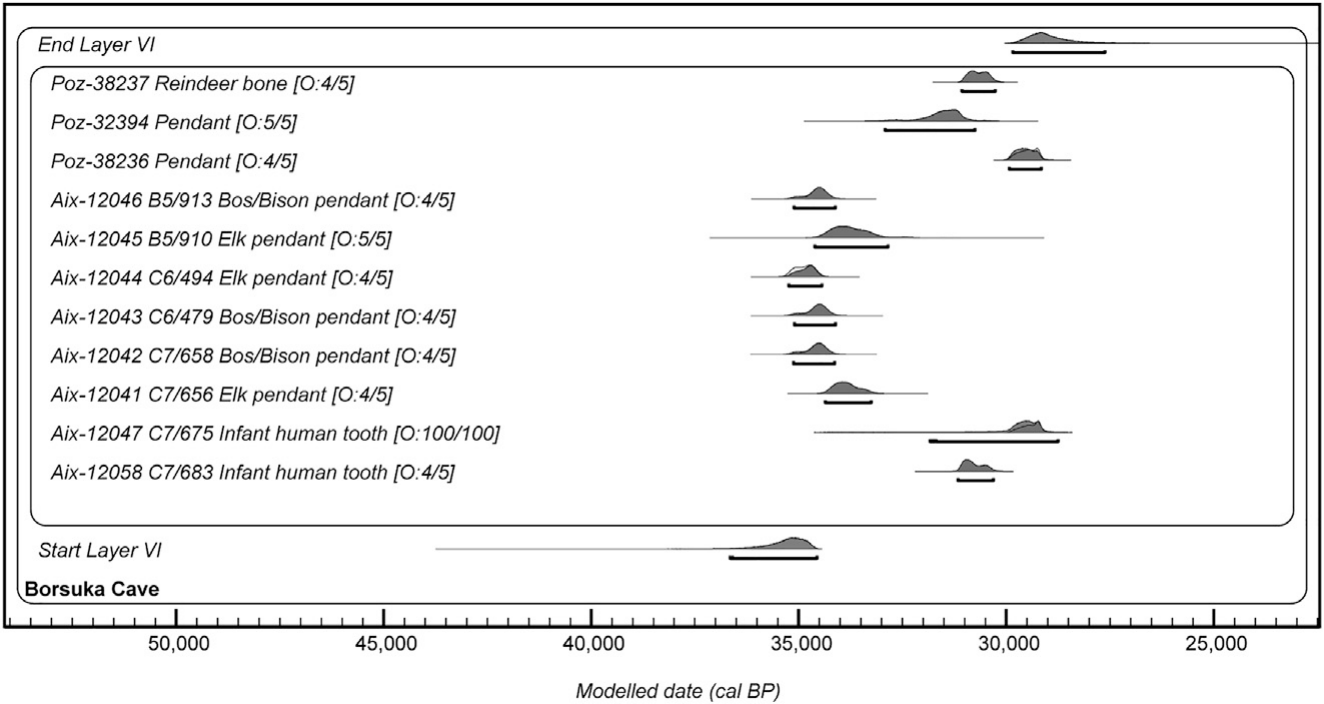
Single phase outlier model showing the posterior probability distributions of the ^14^C dates from Borsuka Cave Layer VI generated in this study (Aix-) and previously (Poz-) The bracket beneath each distribution shows the 95% probability range. The outlier probability (O) is given in the format “[O: posterior outlier probability/prior outlier probability].” The ranges are given in Table S3.

Given the small size of the dentine samples (23.4–67.4 mg) and extracted collagen (1–4.5 mg), we followed a previously established approach for small samples.^41^^,^^42^ Multiple ^14^C dates were obtained from each collagen extract using both graphitization and CO_2_ gas ion source (GIS) methods, and subsequently combined. X^2^ tests were carried out to determine the statistical agreement between replicate dates from the same extract. All of the ^14^C dates obtained fall within the Mid-UP range, from 31,070 ± 770 ^14^C BP to 24,830 ± 290 ^14^C BP, or ∼35,290 to 29,090 cal BP (Figure 5; Table 1; Tables S1 and S2). For the herbivore pendants, all six pendants were dated to 35,290 - 33,260 cal BP (95.4% probability), much older than dates obtained previously on two of the pendants, overlapping with the Late Aurignacian/Early Gravettian periods. For the two human teeth, replicate measurements agree statistically for each collagen extract but the dates from C7/683 (Aix-12058 combined age: 26,610 ± 240 ^14^C BP; 31,160 - 30,320 cal BP at 95.4% probability) are older than C7/675 (Aix-12047 combined age: 25,100 ± 140 ^14^C BP; 29,860 - 29,090 cal BP at 95.4% probability) by ∼1500 ^14^C years (Figure 5; Table S1). When the dates from Layer VI obtained thus far are modeled together in one phase in OxCal, the assemblage spans 6,290-4,770 years, between 36,650 and 27,620 cal BP (Figure 5; Table S3). No dates are available from the overlying and underlying layers to constrain the modeled range, due to a lack of material suitable for dating so none of the dates were identified as outliers. The radiocarbon data is included in Table S1, the background collagen measurements in Table S2 and the modeled ranges of the Borsuka data in Table S3.

### Ancient DNA analysis

Two microsamples between 4.7 and 12.9 mg of dentine powder were drilled from each of the two human teeth (C7/675 and C7/683). The resulting four tooth powder sub-samples underwent DNA extraction, single-stranded library preparation and shotgun sequencing (see method details). Between 1,628,190 and 8,575,605 sequences were generated per library, of which between 2,495 and 37,436 mapped to the human reference genome (hg19^63^) for each library. While no ancient DNA was detected in C7/683, one of the sub-samples from C7/675 was identified as containing ancient DNA based on the observation of between 12.4% and 20.6% C-to-T substitutions on the terminal ends of recovered DNA (Table S4), which is characteristic of ancient DNA.^64^^,^^65^ The duplication rates, or number of times each DNA fragment was sequenced, for these libraries were all around 1 (Table S4), indicating that not all recovered DNA fragments were sequenced. Modern human DNA contamination for libraries produced from this subsample was estimated at 54–56% (Table S4) using the method AuthentiCT^66^ which calculates levels of contamination based on deamination patterns.

**Figure 6:**
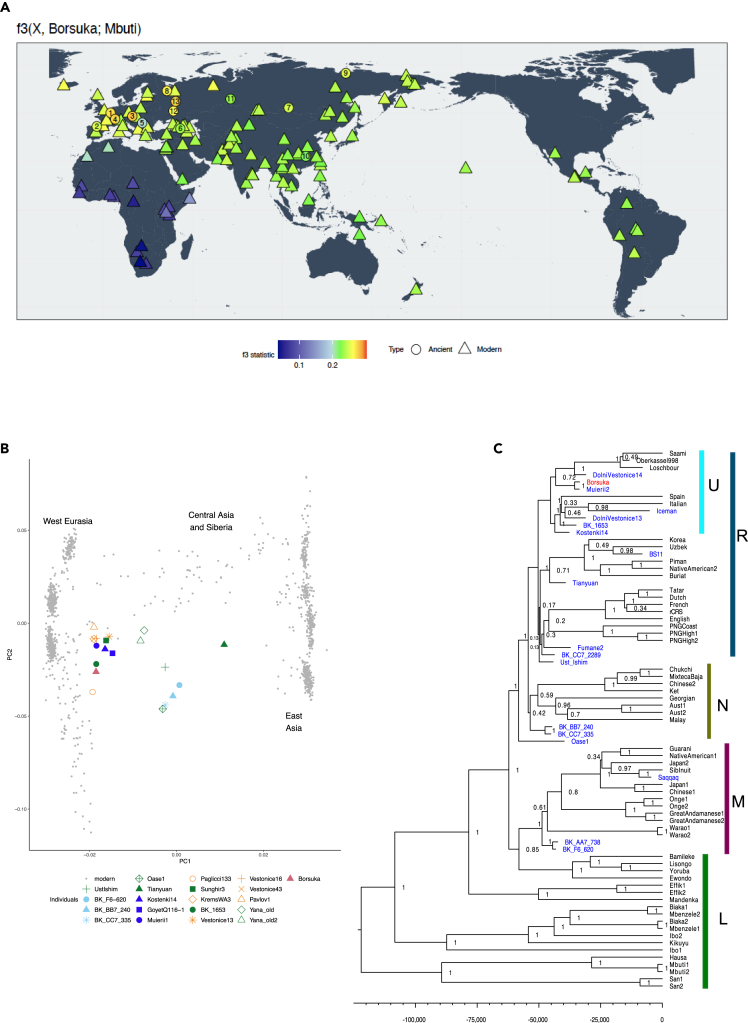
The genetic analysis of the Borsuka C7/675 individual in relation to other modern and ancient humans (A) The calculated allele sharing between the Borsuka individual and a selection of modern and ancient individuals (X) where warmer colors represent higher amounts of shared alleles. The f3 statistic f3(X, Borsuka; Mbuti) was calculated using 28,213 - 31,393 overlapping SNPs for modern populations and 6,697 - 28,642 SNPs for ancient individuals. Ancient individual key: 1- Goyet Q116, 2- EL Miron, 3- Dolní Věstonice16, 4- Bichon, 5- Oase1, 6- Bichon, 7- Mal’ta1, 8- Karelia, 9- Yana2, 10- Tianyuan, 11- Ust-Ishim, 12- Kostenki14, 13- Sunghir3. (B) The projection of 20 ancient individuals onto a principal component analysis of 1,267 modern individuals. Ancient individuals are colored based on their association with the Aurignacian (dark blue), Gravettian (orange), or IUP (light blue) archaeological assemblages or in green if their technological association is uncertain. (C) A subset of the phylogenetic tree generated with BEAST2. Ancient humans are colored in blue (Borsuka in red). Nodes are labeled with their posterior probability. The x axis indicates calibrated years before present.

Despite not detecting ancient DNA in C7/683 to the limits of our resolution, libraries from both teeth were subsequently enriched for human mitochondrial (mt) DNA via hybridization capture,^67^ in an attempt to determine if both likely stem from the same individual (Table S5). Libraries from C7/683 were still included in hybridization captures as the shotgun sequencing was not exhaustive, and the content of nuclear and mitochondrial DNA has been shown to defer in ancient specimens,^68^ leaving the possibility that small amounts of endogenous DNA remained undetected. The enriched libraries contained between 243,078 and 938,984 sequences longer than 34 base pairs that mapped to the human mtDNA revised Cambridge Reference Sequence^69^ with elevated C-to-T substitutions (21.3–35.1% on the 5′ end and 14.5 to 24.6% on the 3′ end). As modern human contamination was detected in each library, ranging from 2.93% to 39.94%, all subsequent analyses were restricted to putatively deaminated fragments that contained a C-to-T substitution within the first three and/or last three terminal bases. Between 15,269 and 60,728 deaminated fragments (corresponding to mtDNA coverages of 46- and 223-fold, respectively) were used to reconstruct near-complete consensus mtDNA genomes. The resulting mtDNA haplotypes from each tooth were the same, consistent with the teeth either being from the same individuals or the same maternal lineage. However, as neither mtDNA genome was complete, we cannot exclude that individually discriminating variants were not detected. The more complete mtDNA genome from C7/675 was then used for tree building and molecular branch shortening (Tables S6 and S7). The resulting molecular date was estimated at 33,533 years BP (95% highest posterior density interval: 28,200 - 38,935 years BP) and the mtDNA genome falls within haplogroup U6 (Figure 6A). Haplogroup U6 is most commonly observed in Northern Africa in present-day humans,^70^^,^^71^ and has previously only been observed in Palaeolithic Europe in the specimens from the site of Peştera Muierii in Romania (∼34,000 years old).^9^^,^^72^^,^^73^ Similar to the Muierii 1 and 2 mtDNA genomes, which were subsequently determined to belong to the same individual,^73^ the Borsuka mtDNA genome is basal to the U6 haplogroup, sharing the 3348G, 10517A, and 16172C positions. Importantly, among the overlapping positions (6 missing positions) no differences are observed between the Muierii 2 and Borsuka mtDNA genomes. Even if both Borsuka teeth and Muierii 2 share an mtDNA genome, this only indicates that they likely lived within ∼2,500 years of each other (Figure S3, method details).

In order to determine the genetic affinity of the child buried in Borsuka Cave to other individuals from the Upper Palaeolithic, we enriched the libraries from C7/675 for ∼1.2 million single nucleotide polymorphisms (SNPs) in the human genome known to be informative for studying human population history.^74^^,^^75^ While the resulting libraries covered 513,237 SNPs, they were also identified as containing approximately 56% modern human contamination (Table S8). This required all downstream analysis to be restricted to the 46,286 putatively deaminated fragments, which were used to determine the sex of the child. As the ratio of autosome, X, Y chromosome SNPs in the 1240k does not follow conventional expectations due to the respective target size, we used an adjustment of the X and Y-rate as has been performed previously with this type of data.^9^ This resulted in an “X-rate” of 0.68 and “Y-rate” of 0.04, consistent with the individual being female (Table 2). When projected onto a principal component analysis (PCA) of modern-day individuals from West Eurasia, Central Asia and East Asia, the Borsuka individual clustered with previously published Upper Palaeolithic Western Eurasians and near modern day Western Eurasians (Figure 6B). The genetic sharing between the Borsuka individual and previously published modern and ancient individuals was calculated with *f3*-statistics, using present-day Mbuti individuals as an outgroup population (Figure 6A). Consistent with the PCA analysis, the greatest affinity among modern day populations was to Western Eurasians. Basal Eurasian ancestry was not detected in the Borsuka individual (Tables S9 and S10, method details). Among ancient individuals, the Borsuka individual shared the most alleles with the ∼35 ka cal BP Bacho Kiro Cave individual (BK1653), ∼34 ka cal BP Muierii 1, ∼31 ka cal BP Věstonice 16 and ∼34 ka cal BP Sunghir 3 individuals when tested with *f3*-statistics (Table S11). When directly comparing the genetic affinity of the Borsuka individual with other ancient individuals with D-statistics, the Borsuka female has a greater affinity to the Gravettian and Aurignacian individuals (Figures S4 and S5; Table S12). However, no significant difference was observed in the affinity of the Borsuka child to the Věstonice vs. Sunghir vs. BK1653 vs. Muierii 1 individuals, precluding the direct association of the Borsuka girl with one of these groups.

**Table 2:** Sex determination based on coverage of nuclear SNPs in the '1240k′ array using putatively deaminated reads

Sample ID	Material	Nyo (# bases covered on Y chromosome)	Nxo (# bases covered on X chromosome)	Nautoo (# bases covered on autosomal chromosomes)	Ny expected (targets on Y chromosome)	Nx expected (targets on X chromosome)	Nauto expected (targets on autosomal chromosomes)	X-ratio (Nxo/Nautoo)/(Nxe/Nautoe)	Y-ratio (Nyo/Nautoo)/(Nye/Nautoe)
C7/675	Human tooth - left udm1	49	1,312	45,032	32,670	49,704	1,150,639	0.674	0.038

A Y-ratio <0.05 indicates a female and >0.2 indicates a male.

## Discussion

The chronological and genetic data presented here confirms the Upper Palaeolithic origin of the infant remains from Borsuka Cave, making these remains currently the oldest female infant burial identified to date. While it is not possible to confirm that the teeth come from the same individual without nuclear DNA, the mitochondrial analysis is consistent with the two teeth belonging to the same individual or the same maternal lineage.

Radiocarbon dating is based on the exponential decay of ^14^C over time. As the concentration of ^14^C in modern carbon is therefore much larger than in ancient samples, any contamination with modern carbon will make ^14^C dates younger than the true age of the sample, with the effects getting progressively worse for more ancient material. Given the ubiquity of modern carbon introduced via the burial environment, handling, storage and analysis of artifacts, when inconsistent dating results are obtained from Palaeolithic material, older ^14^C ages are generally considered more accurate.^29^^,^^76^ The direct date from human tooth C7/683 is older than the date from C7/675. If the two teeth originate from the same individual, this implies that either 1) the older age of C7/683 is correct and the date from C7/675 is an under-estimation of the true age due to the presence of external carbon contamination, or 2) both dates are under-estimations.

Alternatively, the teeth could originate from two infants and both dates could be correct. In addition to the discrepancy in age between the direct human dates and the range of ages from the pendants, this may also be indicated by the difference in δ^15^N values between the teeth and the scatter of the assemblage over several square meters. Given the consistent form of pendant manufacture and that the mtDNA analysis indicates the teeth are at least from maternal relatives (if not from the same individual), a group from the same maternal lineage would need to have used the site for 4,000–6,000 years to account for the dating results in this scenario. The elevated C/N value and additional FTIR peak at ∼2900 cm^−1^ in the C7/675 extract do suggest contamination may have affected the date, and potentially the δ^15^N value. Thus, the most parsimonious interpretation of the results is that the two teeth originate from the same individual and are affected by contamination. If we consider the teeth as originating from one individual, the older date from C7/683 is more likely to be accurate than the younger date from C7/675 as contamination typically decreases the age. Given the small amount of material available for sampling and the C/N values from both teeth of 3.5–3.6, we consider the ∼31.2–30.3 ka cal BP date from C7/683 to be a minimum age for the human remains.

**Figure 7:**
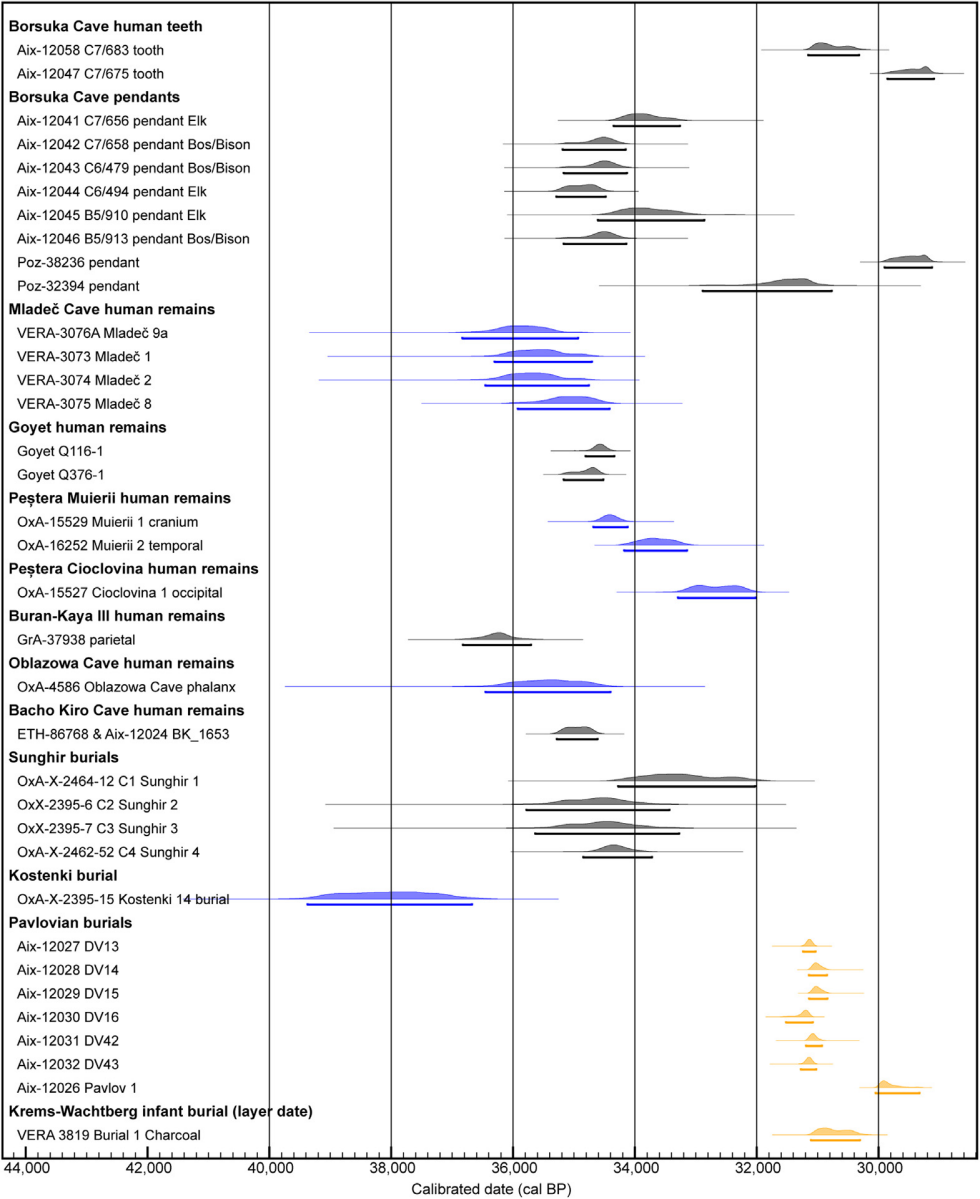
Calibrated radiocarbon dates (95.4% range) from Borsuka Cave infant teeth and pendants compared to UP human burials and human remains in Central and Eastern Eurasia mentioned in the text Human remains tentatively associated with Aurignacian assemblages are shown in blue and Gravettian assemblages in orange while uncertain cultural associations are shown in gray. The Borsuka infant dates are conservatively considered minimum ages.

The older date from C7/683 is contemporaneous with the dates previously obtained from two Borsuka pendants and the ∼31 ka cal BP Pavlovian burials at Dolní Věstonice-Pavlov and Krems-Wachtberg, which would support the association of Borsuka with the Pavlovian culture in Central Europe (Figure 7). However, the new ^14^C dates on the herbivore tooth pendants range in age from 35,290 to 33,260 cal BP (95.4% probability). These new dates for the pendants are older than the ages obtained from the human infant teeth, but also much older than the dates previously obtained on two pendants and a reindeer bone from the same layer (Figure 5).

It is possible that the burial assemblage dates to ∼31 - 30 ka cal BP, and the necklace was constructed from a collection of herbivore teeth of varying age, including some that were pierced and used for the construction of an ornament long after the death of the animal. The solitary lifestyle of European elk (*Alces alces*) implies that the elk teeth were unlikely to have been collected during one event. A recent study of the Sr isotopic compositions and trace element analysis of the enamel from four of the elk tooth pendants (at least 3 individuals) demonstrated a non-local origin of the teeth, indicating that the pendants were transported ∼250 km to Borsuka Cave from an area near the Austria/Slovakia border or northern Hungary, on the southern side of the Western Carpathians.^77^ This evidence for exchange or human regional mobility is supported by Borsuka Cave being the only site in the region containing evidence of the presence of elk ∼27 ka BP (beginning of MIS2) despite favorable environmental conditions.^33^^,^^78^^,^^79^^,^^80^^,^^81^ The presence of Trans-Carpathian raw stone materials in Central Europe also shows high human mobility around the Western Carpathian mountain region.^80^^,^^82^ This evidence suggests that the collection of the herbivore teeth over a period of time is feasible, however, it seems unlikely that the pendants represent animals varying in age by over 5,000 years. Alternatively, low levels of contamination may be affecting some of the ^14^C results with the ∼32 - 29 ka cal BP ages under-estimating the true age of the pendants and human teeth.

The older ∼35.3–33.3 ka cal BP range of dates from the pendants overlap in time with the ∼36.8–34.7 ka cal BP range of directly dated human remains from Mladeč Cave (Czech Republic)^1^ (Figure 7). It has been suggested that the relatively shallow human remains at Mladeč could be the result of intentional deposition,^35^^,^^83^^,^^84^ but the lack of contextual information means this hypothesis cannot be tested. Despite their uncertain context, based on the dates, the Mladeč humans have been associated with Aurignacian artifacts found elsewhere in the cave, including Mladeč-type bone points and pendants made from the teeth of large ungulates (e.g., elk, horse, bison/aurochs).^85^ In comparison to other UP perforated teeth, the Borsuka Cave pendants show close similarity to perforated teeth discovered at Mladeč and generally from Aurignacian sites.^85^^,^^86^^,^^87^ The entire roots of the perforated teeth were heavily scraped on both surfaces, remarkably reducing the root thickness and the perforations were made very close to the root apex by drilling from both sides. The only difference between pendants discovered at Borsuka and Mladeč can be observed in the larger range of species from which teeth were used at Mladeč. Thereby the assemblage from Borsuka cave represents the largest collection of pendants made of large ungulate teeth potentially associated with the Aurignacian culture. Currently, no aDNA is available from the Mladeč individuals to investigate this link further.

While the origin of the Gravettian has been widely debated,^14^ the relatively large collection of burials across Eurasia has provided a significant body of morphological and behavioral data on Gravettian humans across a wide geographical and chronological range. Recent aDNA studies^10^^,^^88^ have demonstrated a genetic distinction between the Gravettian cultures in Central Eastern Europe and Southwestern Europe. The oldest humans discovered to date with genetic continuity to Gravettians and modern West Eurasians are represented by three different genetic ancestry components from the ∼35,000-year-old GoyetQ116-1 individual,^9^ the ∼35,000-year-old individual from Bacho Kiro Cave (BK1653)^89^ and the ∼38,000-year-old Kostenki 14^9,^.^90^ The BK1653 individual is related, but not identical, to the genetic ancestry of GoyetQ116-1 which contributes to the genetic ancestry of the Gravettian individuals in Southwestern Europe, while the former contributes ancestry to the Central European Gravettian individuals. Genetic ancestry from Kostenki 14 has been found in all individuals associated with the Gravettian culture. We aimed to clarify the genetic relationship of the Borsuka individual to individuals of different Palaeolithic cultures, however these associations were limited due to the low amounts of data recovered. The Borsuka individual shares a basal U6 mtDNA haplotype with Peştera Muierii 2, but there is not sufficient nuclear data to determine the full genetic relationship between these individuals in context with other ancient humans. The available data show that the Borsuka individual has the most nuclear genetic affinity to the Bacho Kiro 1653 individual (presumed to be associated with the Aurignacian), as well as individuals from Dolní Věstonice (Gravettian) and Sunghir (early UP). Interestingly, the Borsuka individual is significantly closer to the Bacho Kiro individual than they are to Kostenki 14. This is consistent with Borsuka being an UP Central European, but does not determine a closer affinity to Gravettian or Aurignacian individuals within Central Europe. The ^14^C dates provide a minimum age of 31.3–30.3 ka cal BP for the human remains, which agrees with the molecular date estimation of 33.5 ka that falls roughly at the border between the Late Aurignacian and Early Gravettian periods.

The Borsuka Cave assemblage represents an important data point in the catalog of Palaeolithic human remains for investigating the geographical and chronological range of UP burial practices. Of the very limited number of Upper Palaeolithic infant burials discovered and successfully analyzed with aDNA, this is the first female identified. The previously oldest confirmed female infant burial was found in Mesolithic Italy,^91^ at least 20,000 years after the burial of the Borsuka girl. This study therefore provides the first evidence that child burials incorporating grave goods were not limited to males during the UP. This study further highlights the challenges of working with limited and poorly preserved ancient materials, and emphasizes that employing an interdisciplinary, multi-method approach can still provide valuable insights to allow in-depth investigations of Palaeolithic contexts.

### Limitations of the study

Palaeolithic human remains are rare, and the remains of UP infants even more so. The small size and partial fragmentation of the infant teeth presented an exceptionally limited amount of suitable material for analysis. We aimed to take as little material as possible and therefore limited destructive sampling to two of the six teeth to preserve the morphology of the crowns and ensure their preservation for future studies.

Modern carbon contamination in a ^14^C sample produces an under-estimation of the true age of the material. As sample size decreases, the risk of contamination increases, which is clearly a consideration for the data presented. This is highlighted by the lack of agreement in dates from the two human teeth from the (potentially) same individual and their slightly elevated C/N values, indicating that low levels of C contamination may be present either due to incomplete removal of environmental contaminants during pretreatment or introduced in the laboratory. Given the ubiquity of carbon contaminants in the environment and laboratory, older ages produced from the same sample material are generally considered to be more accurate and reliable. Rigorous compound specific dating approaches can be employed to isolate endogenous hydroxyproline in bone collagen to overcome contamination issues.^27^^,^^92^^,^^93^ However, the small proportion of hydroxyproline in collagen means this approach requires large starting sample sizes, thus making the approach not feasible for this study. As contamination of the human dates is indicated we therefore consider them to be minimum ages. The molecular dating technique (calibrated using other directly radiocarbon dated human remains) indicates an age of ∼33.5 ka BP (albeit with a very large confidence interval), which supports the ∼31 ka cal BP being a minimum age. The new data presented nevertheless confirm the UP origin of the human remains. Given the challenges and the limited data available, we cannot conclude a definitive age for the burial assemblage or a more definitive association with a specific Palaeolithic culture. However, even using very small sample sizes for dating (in the order of aDNA sample sizes), we confirm the placement of the assemblage around the border of the Late Aurignacian/Early Gravettian, supported by the aDNA analysis and the typology of the pendants.

The high levels of modern human contamination and limited amount of endogenous DNA present in both infant teeth limited the power of determining different genetic affinities beyond broad population genetic relationships. This resulted in reduced coverage of both the mtDNA and nuclear genomes. As shown in Figures S6–S9, decreased amounts of data result in missing some genetic affinities. It is likely that with more data it would have been possible to deconvolute the association of the Borsuka girl to Bacho Kiro 1653, or the Věstonice and Sunghir clusters, but unfortunately the limited data prevented this.

The limitations arising due to contamination emphasize the importance of taking efforts to minimize the contamination of archaeological materials. This includes wearing gloves and face masks during excavation and handling of human remains and artifacts, appropriate storage and consideration of sampling prior to consolidants or glues being applied.

## STAR★Methods

### Key resources table

**Table:** 

REAGENT or RESOURCE	SOURCE	IDENTIFIER
**Biological samples**		

Osteological remain	This study	C7/675
Osteological remain	This study	C7/683
Osteological remain	This study	C7/656
Osteological remain	This study	C7/658
Osteological remain	This study	C6/479
Osteological remain	This study	C6/494
Osteological remain	This study	B5/910
Osteological remain	This study	B5/913

**Chemicals, peptides, and recombinant proteins**		

0.5M EDTA pH 8.0	AppliChem	Cat.no. A4892
100% Tween 20	Merck	Cat.no. P5927-100ML
10 mg/mL Proteinase K	Sigma Aldrich	Cat.no. P6556
H20, HPLC grade	Merck	Cat.no. 270733
Guanidine hydrochloride	Sigma Aldrich	Cat.no. G3272
Isopropanol	Merck	Cat.no. 109634
100% Ethanol	Merck	Cat.no. 100983
PE Buffer	Qiagen	Cat.no. 19065
1 M Tris-HCl pH 8.0	AppliChem	Cat.no. A4577
3M Sodium Acetate, pH 5.2	Merck	Cat.no. S7899-500ML
5 M NaCl	Merck	Cat.no. S5150-1L
20% SDS	Thermo Fisher Scientific	Cat.no. AM9820
10x AmpliTaq Gold buffer (without Mg)	Life Technologies	Cat.no. 4379874
1M NaOH	Roth	Cat.no. 9062.3
20x SSC	Thermo Fisher Scientific	Cat.no. AM9763
Silica magnetic beads	G-Biosciences, VWR	Cat.no. 786-915
T4 RNA ligase reaction buffer including 50% (wt/vol) PEG 8000	NEB	Cat.no. B0216L
ATP solution (100 mM)	Thermo Fisher Scientific	Cat.no. R0441
T4 polynucleotide kinase 10 U uL^−1^	Thermo Fisher Scientific	Cat.no. EK0031
T4 DNA ligase high concentrated 30 U uL^-1^	Thermo Fisher Scientific	Cat.no. EL0013
Fast AP (1U/uL)	Thermo Fisher Scientific	Cat.no. EF0651
Klenow fragment (10 U/uL), including 10X reaction buffer	Thermo Fisher Scientific	Cat.no. EP0052
T4 DNA Ligase, 5 U/uL, including 10X reaction buffer and 50% (wt/vol) PEG-4000	Thermo Fisher Scientific	Cat.no.EL0012
Dynabeads MyOne Streptavidin C1 beads	Life Technologies	Cat.no. 65001
PEG-8000 powder	Promega	Cat.no. V3011
Sera-Mag SpeedBeads	Sigma Aldrich	Cat.no. GE6515-2105-050250
UltraPure DNase/RNase-Free Distilled Water	Invitrogen	Cat.no. 10977049
dNTPs 25mM each	Thermo Fisher Scientific	Cat.no. R1121
Herculase II Fusion DNA polymerase, including 5X Herculase II reaction buffer	Agilent	Cat.no. 600679
Human Cot-1 DNA	Thermo Fisher Scientific	Cat.no. 15279011
2x HI-RPM hybridization buffer, including Agilent blocking agent (dissolve in 1250uL water)	Agilent	Cat.no. 5188-5380_25 mL
Maxima Probe qPCR Master Mix (2x)	Thermo Fisher Scientific	Cat.no. K0261
AccuPrime *Pfx* DNA polymerase, including 10x AccuPrime reaction mix	Thermo Fisher Scientific	Cat.no. 12344-024
Maxima SYBR Green qPCR Master Mix (2X)	Thermo Fisher Scientific	Cat.no. K0253
Ultrapure H_2_O (resistivity value: 18.2 MΩ·cm @ 25 °C; ≤ 5 ppb)	MilliporeSigma	MilliQ Element A10 Water Purification system
HCl 37%	Roth	Nr. 9277.1
HCl (6N)	Roth	Nr. 0281.1
NaOH (2N)	Roth	Nr. T135.1
Ezee Filters 9ml	Elkay Labs	Cat.no. 127-3193-000
Vivaspin Turbo 15 Ultrafilters PES (30 kDa MWCO)	Sartorius	Cat.no. VS15T01
AMS standard reference material Oxalic acid	NIST	SRM 4990C
IAEA-CH-6 (EA-IRMS standard)	IAEA	https://nucleus.iaea.org/sites/ReferenceMaterials/Pages/IAEA-CH-6.aspx
IAEA-CH-7 (EA-IRMS standard)	IAEA	https://nucleus.iaea.org/sites/ReferenceMaterials/Pages/IAEA-CH-7.aspx
IAEA-N-1 (EA-IRMS standard)	IAEA	https://nucleus.iaea.org/sites/ReferenceMaterials/Pages/IAEA-N-1.aspx
IAEA-N-2 (EA-IRMS standard)	IAEA	https://nucleus.iaea.org/sites/ReferenceMaterials/Pages/IAEA-N-2.aspx
Methionine	Elemental Microanalysis, Okehampton, UK	B2100

**Critical commercial assays**		

Custom capture probes	Agilent	N/A
P5 8bp primer plate (384)	Eurogentec	N/A
P7 8bp primer plate (384)	Eurogentec	N/A
MinElute PCR Purification kit	Qiagen	Cat.no. 28006
QIAquick Nucleotide Removal kit	Qiagen	Cat.no. 28306

**Deposited data**		

European Nucleotide Archive (nuclear DNA data)	This study	PRJEB66365
Dryad (mtDNA genome)	This study	https://doi.org/10.5061/dryad.47d7wm3m4

**Oligonucleotides**		

(ddC, dideoxycytidine; TEG, triethylene glycol spacer; ∗, phosphothioate linkage; [N], 2′-O-methyl-RNA; {N}, locked nucleic acid (LNA); SpacerC12, 12-carbon spacer; FAM, fluorescein amidite; BHQ1, Black Hole Quencher-1)		
TL 181 100uM (desalted), 1^st^ adapter Phosphate-AGATCGGAAGAAA[A][A][A][A][A][A][A]-TEG-Biotin	IDT	N/A
TL 159 100uM (desalted), splinter SpacerC12-[A][A][A]CTTCCGATCTNNNNNNNN[A]-AminoC6	Eurogentec	N/A
CL 128 100uM (HPLC), extension primer GTGACTGGAGTTCAGACGTGTGCTCTTCC∗G∗A∗T∗C∗T	Eurogentec	N/A
CL 53 500uM (HPLC), 2^nd^ adapter strand 1 CGACGCTCTTC-ddC	Sigma-Adrich (Merck)	N/A
TL 178 500uM (desalted), 2^nd^ adapter strand 2 Phosphate-GGAAGAGCGTCGTGTAGGGAAAGAGTGTA	Eurogentec	N/A
CL 304 100uM (HPLC), control DNA Phosphate-ATTCAGCTCCGGTTCCCAACGATCAAGGC GAGTTACATGA-Phosphate	Sigma-Adrich (Merck)	N/A
IS5 100uM (desalted), forward primer AATGATACGGCGACCACCGA	Sigma-Adrich (Merck)	N/A
IS5 biotinylated 100uM (desalted), forward primer Biotin-AATGATACGGCGACCACCGA	Sigma-Adrich (Merck)	N/A
IS6 100uM (desalted), reverse primer CAAGCAGAAGACGGCATACGA	Sigma-Adrich (Merck)	N/A
CL105 100uM (desalted), forward primer ACACTCTTTCCCTACACGACGCTCTTCCTCGTCGTTT GGTATGGCTTC	Sigma-Adrich (Merck)	N/A
CL106 100uM (desalted), reverse primer GTGACTGGAGTTCAGACGTGTGCTCTTCCGATCTTC ATGTAACTCGCCTTGATCGT	Sigma-Adrich (Merck)	N/A
IS7 100uM (HPLC), forward primer A&B ACACTCTTTCCCTACACGAC	Sigma-Adrich (Merck)	N/A
IS8 100uM (HPLC), reverse primer A GTGACTGGAGTTCAGACGTGT	Sigma-Adrich (Merck)	N/A
IS10 100uM (reverse phase - HPLC), probe A FAM-A{G}A{T}C{G}GAAGAGC{A}CAC-BHQ1	Eurogentec	N/A
CL107 100uM (HPLC), reverse primer B TCATGTAACTCGCCTTGATCGT	Sigma-Adrich (Merck)	N/A
CL118 100uM (HPLC), probe B FAM-TTCAGCTCCGGTTCCCAACGAT-BHQ1	Sigma-Adrich (Merck)	N/A

**Software and algorithms**		

leeHom	Renaud et al.^94^	https://bioinf.eva.mpg.de/leehom/
BWA version 0.5	N/A	https://github.com/mpieva/network-aware-bwa
SAMtools version 1.3.1	Li, H. et al.^95^	http://www.htslib.org/doc/1.3.1/samtools.html
Bam-rmdup version 0.6.1	NA	https://github.com/mpieva/biohazard-tools
MAFFT v.7	Katoh, K. et al.^96^	https://mafft.cbrc.jp/alignment/server/index.html
BEAST V2.6.6	Bouckaert, R. et al.^97^	http://www.beast2.org/
MODEL_SELECTION	Leaché, A.D. et al.^98^	https://beast.community/workshop_model_selection
Tracer v1.7	Rambaut, A. et al.^99^	https://beast.community/tracer
Bam-caller v0.1		https://github.com/bodkan/bam-caller
Smartpca	Patterson, N. et al., 2006 and Price, A.L. et al., 2006^100^^,^^101^	https://reich.hms.harvard.edu/software
ADMIXTOOLS	Patterson, N. et al.^102^	https://reich.hms.harvard.edu/software
Admixr v0.7.1	Petr, M. et al.^103^	https://github.com/bodkan/admixr
OxCal v4.4	Bronk-Ramsey,^104^	https://c14.arch.ox.ac.uk/oxcal.html
BATS v3.64	Wacker et al.^105^	
AGE3 software v4.666	IonPlus AG	www.ionplus.ch
GIS software v4.15	IonPlus AG	www.ionplus.ch

**Other**		

Allen ancient DNA resource (aadr) for previously published ancient and modern DNA data	N/A	https://reich.hms.harvard.edu/allen-ancient-dna-resource-aadr-downloadable-genotypes- present-day-and-ancient-dna-data
IntCal20 Calibration curve	Reimer et al.^106^	

### Resource availability

#### Lead contact

Further information and requests for resources and reagents should be directed to and will be fulfilled by the lead contact, Helen Fewlass (helen.fewlass@crick.ac.uk).

#### Materials availability

This study did not generate new unique reagents.

#### Data and code availability

• Nuclear DNA data have been deposited at the ENA (European Nucleotide Archive) repository: PRJEB66365 and mitochondrial DNA data at the Dryad repository: https://doi.org/10.5061/dryad.47d7wm3m4. Both are publicly available as of the date of publication. The accession number and DOI are also listed in the key resources table. This paper also analyses existing, publicly available data from the Allen Ancient DNA Resource. The link to the dataset is listed in the key resources table. The radiocarbon data is available in this paper’s supplemental information.
• All original code is available in this paper’s supplemental information.
• Any additional information required to reanalyse the data reported in this paper is available from the lead contact upon request.

### Experimental model and subject details

#### Archaeological information

Three Elk and three Bos/Bison incisor tooth pendants were selected for dating from squares B5, C6 and C7 to represent the spread of the 112 pendants across the trench, each originating from a different animal based on tooth morphology. Five of the six teeth selected had pre-existing broken roots with only part of the drilled hole preserved. The two human teeth C7/675 (uldm1) and C7/683 (fragment of a cusp of a deciduous molar) were selected for analysis as they had the largest amount of dentine preserved and were microCT-scanned prior to the study to preserve their 3D morphology. Following excavation (2009-2010), the material was washed and wet-sieved in a local stream and handled without gloves prior to laboratory analysis, as per standard archaeological procedure. No consolidants were applied to the surface of the human or herbivore teeth.

### Method details

#### Radiocarbon dating

##### Pretreatment for radiocarbon dating

Small samples of the dentine (human: 23.4 - 41.1 mg; herbivore: 34.4 - 67.4 mg) were removed using a dentist’s rotary drill. Dentine samples were pretreated in the Human Evolution department at the Max Planck Institute for Evolutionary Anthropology, Leipzig, using a previously published protocol for small samples.^41^ Briefly, sample chunks were demineralised in HCl 0.5M at 4**°**C until mechanically soft and CO_2_ effervescence stopped. Samples were treated with NaOH 0.1M to remove humic acid contamination and re-acidified in HCl 0.5M. The samples were gelatinised in HCl pH3 for 3-10 h at 70**°**C. The gelatin extracts were then filtered (Ezee-filters, Elkay labs, UK) to remove >80 μm particles and ultrafiltered to concentrate the >30 kDa weight fraction. Filters were pre-cleaned according to Bronk-Ramsey et al.^107^ Samples were rinsed to a neutral pH between each step. The extracts were freeze-dried for >48 hours and weighed immediately to determine the collagen yield. Small aliquots of bones dating beyond the ^14^C method (background) were pretreated and measured alongside the samples.

##### Collagen quality assessment

To assess the quality of the extracts, ∼0.4 mg collagen was weighed into a tin capsule and measured on a ThermoFinnigan Flash 2000 elemental analyser (EA) coupled to a Thermo Delta plus XP isotope ratio mass spectrometer (IRMS) via a Conflo III interface (Thermo Fisher Scientific, Germany) to determine the elemental (C%, N%, C/N) and stable isotopic values (δ^13^C, δ^15^N). Stable carbon isotope ratios were expressed relative to Vienna PeeDee Belemnite (VPDB) and stable nitrogen isotope ratios were measured relative to atmospheric N_2_ (AIR), using the delta notation (δ) in parts per thousand (‰). Stable isotope delta values were two-point scale normalised using international reference material IAEA-CH-6 (sucrose, δ^13^C = -10.449 ± 0.033 ‰), IAEA-CH-7 (polyethylene, δ^13^C = -32.151 ± 0.050 ‰), IAEA-N-1 (ammonium sulphate, δ^15^N = 0.4 ± 0.2 ‰) and IAEA-N-2 (ammonium sulphate, δ^15^N = 20.3 ± 0.2 ‰). Repeated analysis of internal and international standards indicates an analytical error of ± 0.2‰ (1σ). Two in-house quality control standards were used to quality check the scale normalisation and evaluate analytical precision: 1) EVA-0012 methionine (Elemental Microanalysis, Okehampton, UK), n = 12, δ^13^C = -28.0 ± 0.14 ‰ (1 s.d.), δ^15^N = -6.5 ± 0.12 ‰ (1 s.d.); and EVA MRG pig gelatin, n= 11, δ^13^C = -19.6 ± 0.24‰ (1 s.d.) and δ^15^N = 4.9 ± 0.11 ‰ (1 s.d.). These values compare well with the long-term average values of δ^13^C = -28.0 ± 0.1‰ (1 s.d.) and δ^15^N = -6.4 ± 0.1‰ (1 s.d.) for EVA-0012 and δ^13^C = -19.7 ± 0.3‰ (1 s.d.) and δ^15^N = 5.0 ± 0.1‰ (1 s.d.) for EVA MRG.

Sufficient collagen was available from C7/675, C7/658, C6/494 for additional quality checks using Fourier Transform Infrared Spectroscopy (FTIR).^54^^,^^55^^,^^56^ Roughly 0.3 mg collagen was homogenised and mixed with ∼40 mg of IR grade KBr powder in an agate mortar and pestle. The powder was pressed into a pellet using a manual hydraulic press (Wasserman) and analysed with an Agilent Technologies Cary FTIR with a DTGS detector. Spectra were recorded in transmission mode at 4 cm^−1^ resolution with averaging of 34 scans between 4000 and 400 cm^−1^ using Resolution Pro software (Agilent Technologies) and were compared to library spectra of well preserved collagen.

##### AMS dating

The extracts were dated on the AixMICADAS^108^ installed at CEREGE (Centre de Recherche et d'Enseignement de Geosciences de l'Environnement) in Aix-en-Provence, France. Each collagen extract was split and measured in several aliquots as per the protocol for small collagen extracts described in Fewlass et al.^41^^,^^42^ (Table S1). Where sufficient material was available, collagen (combustion weight ∼700 ug C) was weighed into aluminium cups and graphitized using the AGE 3^109^ (Automated Graphitisation Equipment) prior to dating on the AixMICADAS. Oxalic acid II standards and background collagen samples (Table S2) were measured in the same session and used in the age calculation of the archaeological samples in BATS.^105^ A relative error of 30% was applied to the blank value and an additional uncertainty of 1.6‰ was propagated in the F^14^C error calculation as per standard practice.

Gas measurements were performed using the protocol described in Tuna et al.^110^ and Fewlass et al.^41^ Small aliquots of collagen (combustion weight ∼60 ug C) were introduced into cleaned silver cups (800°C, 2 h) and combusted in an Elementar Vario MICRO cube EA (Elementar Analysensysteme GmbH, Germany) which was directly coupled to the gas ion source (GIS) of the AixMICADAS.^111^^,^^112^ Oxalic acid II NIST standards (from a gas canister) were measured to normalize and correct samples for fractionation. The age of each sample was corrected with background measurements from the same batch (Table S2). A relative error of 30% was applied to the blank value and an additional uncertainty of 4‰ was propagated in the F^14^C error.

#### Ancient DNA analysis

##### Data generation and shotgun sequencing

Sampling of the teeth for radiocarbon dating and genetic analysis was performed in a dedicated clean room at the Max Planck Institute for Evolutionary Anthropology. A microsampling approach was used for sample collection from the two of the infant teeth (C7/675 and C7/683) for downstream genetic analyses. Between 7.2 and 12.9 mg of dentine was collected from the two roots of C7/675 and two microsamples of 4.7 and 5.2 mg of dentine were drilled from the crown of C7/683 for a total of four samples. To extract DNA from the tooth powder, 500 uL of lysis buffer (for 5 mL lysis buffer: 4.5 mL 0.5M EDTA pH 8.0, 2.5 uL of Tween 20,125 uL of 10mg/mL proteinase K, 373 uL water) was added to each tooth powder aliquot for overnight lysis at 37**°**C. The resulting lysate was purified following the automated extraction protocol described,^113^ using binder buffer ‘D’. Each DNA extract was then converted into dual-indexed single-stranded libraries^106^ and sequenced on Illumina MiSeq or HiSeq2500 platforms in pools of 63-96 libraries that included libraries from other projects not discussed in this paper. Negative controls were included starting at both the DNA extraction and library preparation steps. Base calling was performed using Bustard (Illumina) and *leeHom*^104^ was used to trim adapters and merge overlapping forward and reverse sequences. The resulting sequences were mapped to the human reference genome (hg19^63^) using BWA version 0.5 (https://github.com/mpieva/network-aware-bwa) with ancient DNA parameters (-n 0.001 -o 2 -l 16500). Reads that were not a perfect match to expected index combinations, shorter than 35 base pairs, or had a mapping quality less than 25 were removed using SAMtools version 1.3.1^104^. *Bam-rmdup* version 0.6.1 (https://github.com/mpieva/biohazard-tools) was used to remove PCR duplicates. Each library (including controls) was evaluated for the presence of ancient human DNA based on the presence of elevated C-to-T substitutions on the terminal ends of DNA fragments (Table S4). Potential cross-contamination due to index swapping between libraries was evaluated using a previously published method^67^ that evaluates all sequenced index pairs in a sequencing run to estimate the number of reads assigned to a library that may originate from another library. All libraries were estimated to have less than 6 cross-contaminated reads. The negative controls were also examined for evidence of ancient DNA based on elevated C-to-T substitutions and no evidence of aDNA was identified in these libraries (Table S4).

### Quantification and statistical analysis

#### Radiocarbon data analysis and reporting

The radiocarbon dates were calibrated and analysed using OxCal 4.4^114^ and the IntCal20 calibration curve.^115^ The dates were input into a one phase General t-type Outlier Model with a 5% prior likelihood of being an outlier, except C7/675 which was given an outlier likelihood of 100% due to the C/N value and FTIR analysis^116^ (Table S3). As per convention (e.g. Stuiver & Polach,^94^ Millard^95^), throughout the text uncalibrated ^14^C ages are reported with the abbreviation ‘BP’, meaning ‘radiocarbon years before AD 1950’ and are reported with 1σ errors, whereas calibrated ranges are denoted as ‘cal BP’ through the text and are given at the 95.4% (2σ) probability range. The agreement of the replicate dates from a single extract was tested using a X^2^ test using the R_Combine function. All previously reported dates have been re-calibrated with the IntCal20 curve.^115^

#### Mitochondrial DNA analysis

Six libraries (three from the samples and three negative controls) were enriched for human mitochondrial DNA via hybridization capture on an automated platform as described in^67^ and sequenced on Illumina’s MiSeq platform in pools of 32 to 76 libraries. Analysis of the resulting sequences was performed as described for the shotgun sequencing with the exception that libraries were mapped to the rCRS (Table S5). The reconstruction of mitochondrial genomes was performed on one library from each C7/675 and C7/683 where a coverage of at least 5-fold and support of at least 80% was required for calling a consensus base using only putatively deaminated reads.

No evidence of cross-contamination from index swapping between libraries (< 1 contaminating read per library) was identified in the sequencing runs. Reads with elevated C-to-T substitutions were also absent from the negative controls (Table S5). While the library negative controls had relatively few unique mtDNA reads (5 to 36) there were 680 unique mtDNA reads in the extraction negative control. Support for the variants observed in C7/675 and C7/683 were examined in the extraction negative control. The only shared variant was a G at position 73, which is a common variant across human populations,^117^^,^^118^ supporting that laboratory contamination did not impact downstream analyses.

The resulting mtDNA genome from C7/675 was aligned to the rCRS with mtDNA genomes from one Neanderthal (Vindija33.19), 54 present day^119^ and 18 ancient humans (Table S6) with MAFFT^96^ for tree building and molecular dating using the program BEAST V2.6.6^97^. Present-day individuals were set to 0 and the Borsuka individual was constrained to an age range of 0 to 60,000. A model using a strict clock and constant population was selected after using the path sampling approach with 40 path steps from the MODEL_SELECTION^98^ package in BEAST2 to evaluate different clock and tree models (Table S7). These models were selected because there was no significant difference identified between the models and therefore the simplest were selected. In this testing a chain length of 25 million iterations, 0.3 alpha parameter, pre-burn of 75,000 iterations, and an 80% whole chain burn-in were used. For both testing and the final analysis, clock models were set with a normal distribution around a mean mutation rate of 2.67E-08 per base per year^120^ with a sigma of 1.00E-10.^121^ The Tamura-Nei 193 (TN93)^122^ substitution model was used for all models. For the final tree building and molecular dating, 30 million iterations (1 million pre-burn iterations) of three Markov chain Monte Carlo runs with sampling every 2,000 trees were combined using Logcombiner2 from BEAST2. The tree annotator program from BEAST2 was used to identify a single tree from the combined tree file, which was visualised with Figtree v1.4.4. The Tracer v1.7^119^ program was used to examine the resulting molecular dates.

#### Estimating TMRCA for two mtDNA genomes with no differences

In order to estimate the potential time span between two mtDNA genomes that share an mtDNA haplotype, we followed a previously published approach^100^ to estimate the time to the most recent common ancestor (TMRCA) in this scenario. Following Skov et al., 2022,^100^ we used a probability density (see below), which combines equations 7, 8, and 9 from Tavare et al., 1996^101^ to determine the mean coalescence time for two sequences with 0 differences. To calculate *Θ* we used a mutation rate (***μ***) of 2.67e-8 mutations per base pair per year, a generation time of 29 years,^102^ length of 15,569, and set the effective population size to 1,000 individuals (*Θ*=2∗N∗***μ***∗L). As the resulting mean time (Figure S3) represents one branch length, the total branch length is then 2 ∗ 1,206 years, or 2,412 years.

$$Probability\;density\;function\text{:}f_{coalescence\;time}\left(t|k\right)=\frac{{\left(1+ϴ\right)}^{1+k}}{k!}t^{k}e^{-\left(1+ϴ\right)t}$$

#### Nuclear DNA analysis

*Twelve* libraries (six samples and six negative controls) were also enriched for ∼1.2 million SNPs (‘1240k array’) previously described in^74^ and^75^ via hybridization capture. They were sequenced on Illumina’s MiSeq and NextSeq platforms and analysed as described in the shotgun sequencing section (Table S8). For these libraries less than 0.001% of total reads (<15 reads) were estimated as originating from index swapping. The data from the two libraries from C7/675 identified as containing ancient DNA were merged for downstream analysis using samtools.^123^ Pseudo-haplotype genotyping was performed using random read sampling with bam-caller (https://github.com/bodkan/bam-caller; v0.1) after trimming the three termini bases of each fragment to minimise the effects of deamination on downstream analyses. These data were converted into EIGENSTRAT format and merged with previously published genotypes of ancient and modern humans previously published and compiled (https://reich.hms.harvard.edu/allen-ancient-dna-resource-aadr-downloadable-genotypes- present-day-and-ancient-dna-data). The principal component analysis was performed using *smartpca*^103^^,^^124^ on the 597,573 SNPs in the Affymetrix Human Origins array^125^ on 1,395 modern-day humans from West Eurasia, the Americas, Central Asia and Siberia, and East Asia. The Borsuka individual was then projected onto the PCA with 22 ancient individuals. The genetic relationship between the Borsuka individual and modern and ancient individuals was explored further with SNPs from the ‘1240k’ array using ADMIXTOOLS^125^ with the R package *admixr* v0.7.1.^126^ The modern populations used were from Simons Genome Diversity Project.^127^

The high amount of modern human contamination in the genetic data recovered from C7/675 required all downstream analysis to be restricted to putatively deaminated fragments only. This limited the data to only 46,286 SNPs in the ‘1240k’ panel. As this only represents 3.8% of the SNPs within the panel, this raises concern as to what conclusions are possible to draw with this limited amount of data. Therefore, comparisons between other ancient humans and the Borsuka individual were restricted only to individuals with at least 500,000 SNPs with one exception. The Věstonice complex includes multiple individuals with varying qualities of genetic data. Four of these individuals were included for analysis to serve as a proxy for Borsuka for the input of limited data on each analysis.

##### Basal Eurasian ancestry evaluation

D-statistics was used to explore how the Borsuka individual’s genetic affinities compared to other previously published ancient humans. It has previously been shown that the D statistic is ∼ 0 for both D(Kostenki14, X; Han/Dai, Mbuti) and D(W, Stuttgart; Han, Mbuti), when W/X is a European from ∼39,000 to 14,000 years ago without Basal Eurasian ancestry^[9]^. Calculating these D-statistics with Borsuka in the X or W position is consistent with an individual that also does not contain basal Eurasian ancestry (Tables S9 and S10).

## Supplemental information

Document S1. Figures S1–S9 and Tables S9–S12

Table S1. Sample pretreatment information and 14C dating of tooth dentine from Borsuka Cave, related to Figures 5 and 6

Table S2. AMS measurements from background (R-EVA 1753 and 2907) and standard (R-EVA 123) bones measured alongside Borsuka Cave samples and used in their age calculation, related to STAR Methods (AMS dating)

Table S3. Output of general outlier model of Borsuka Cave Layer VI dates, relating to Figure 5

Table S4. Summary of results from shotgun sequencing for initial screening of samples for ancient DNA content, related to STAR Methods (Ancient DNA: Data generation and shotgun sequencing)

Table S5. Summary of results from sequencing of libraries enriched for mitochondrial DNA, related to STAR Methods (Ancient DNA: Mitochondrial DNA analysis)

Table S6. Previously published ancient individuals included for tree building and molecular dating, related to Figure 6C

Table S7. The marginal log likelihood for different tree and clock model combinations, related to STAR Methods (Ancient DNA: Mitochondrial DNA analysis)

Table S8. Summary of results from sequencing of libraries enriched for nuclear SNPs in the '1240k′ array, related to STAR Methods (Ancient DNA: Nuclear DNA analysis)
