# Supplementary material for: Chronological and genetic analysis of an Upper Palaeolithic female infant burial from Borsuka Cave, Poland
Source: iScience. 2023 Oct 23;26(12):108283. doi: 10.1016/j.isci.2023.108283 (PMC10690573; doi:10.1016/j.isci.2023.108283)
Supplement: Document S1. Figures S1–S9 and Tables S9–S12 [file mmc1.pdf]

iScience, Volume *26*

Supplemental information

Chronological and genetic analysis of an Upper Palaeolithic female infant burial from Borsuka Cave, Poland

Helen Fewlass, Elena I. Zavala, Yoann Fagault, Thibaut Tuna, Edouard Bard, Jean-Jacques Hublin, Mateja Hajdinjak, and Jarosław Wilczyński

Supplementary Information

CQL code for OxCal (4.4), related to site model in Figure 5 and Supplementary Table 3:

```
Plot()
 {
  Outlier_Model("General",T(5),U(0,4),"t");
  Sequence("Borsuka Cave")
  {
   Boundary("Start Layer VI");
   Phase("Layer VI")
   {
    R_Combine("Aix-12058 C7/683 Infant tooth")
    {
     R_Date("12058.1.1", 26610, 410);
     R_Date("12058.1.2", 26960, 430);
     R_Date("12058.1.3", 26260, 400);
     Outlier(0.05);
    };
    R_Combine("Aix-12047 C7/675 Infant tooth")
    {
     R_Date("12047.1.2", 25140, 150);
     R_Date("12047.1.1", 24830, 350);
     Outlier(1.0);
    };
    R_Combine("Aix-12041 C7/656 Elk pendant")
    {
     R_Date("Aix-12041.1.2", 29180, 250);
     R_Date("Aix-12041.1.1", 30150, 690);
     Outlier(0.05);
    };
    R_Combine("Aix-12042 C7/658 Aurochs pendant")
    {
     R_Date("Aix-12042.1.2", 29940, 280);
     R_Date("Aix-12042.1.1", 31070, 770);
     Outlier(0.05);
    };
    R_Combine("Aix-12043 C6/479 Aurochs pendant")
    {
     R_Date("Aix-12043.1.2", 29990, 280);
```

```
   R_Date("Aix-12043.1.1", 30570, 730);
   Outlier(0.05);
  };
  R_Combine("Aix-12044 C6/494 Elk pendant")
  {
   R_Date("Aix-12044.1.2", 30070, 280);
   R_Date("Aix-12044.1.3", 30740, 280);
   R_Date("Aix-12044.1.1", 30930, 760);
   Outlier(0.05);
  };
  R_Combine("Aix-12045 B5/910 Elk pendant")
  {
   R_Date("Aix-12045.1.1", 28760, 620);
   R_Date("Aix-12045.1.2", 29310, 640);
   R_Date("Aix-12045.1.3", 29840, 670);
   Outlier(0.05);
  };
  R_Combine("Aix-12046 B5/913 Aurochs pendant")
  {
   R_Date("Aix-12046.1.2", 30090, 280);
   R_Date("Aix-12046.1.1", 30030, 680);
   Outlier(0.05);
  };
  R_Date("Poz-38236 Pendant", 25150, 160)
  {
   Outlier(0.05);
  };
  R_Date("Poz-32394 Pendant", 27350, 450)
  {
   Outlier(0.05);
  };
  R_Date("Poz-38237 Reindeer", 26430, 180)
  {
   Outlier(0.05);
  };
  Span("Span of Layer VI");
  Date("Duration Layer VI");
 };
 Boundary("End Layer VI");
};
};
```

Supplementary Tables

W	X	Z	Y=Dai D	Y=Dai stderr	Y=Dai Zscore	Y=Dai nsnps	Y=Han D	Y=Han stderr	Y=Han Zscore	Y=Han nsnps
Kostenki14	Oase	Mbuti	0.0265	0.006864	3.856	191,181	0.0237	0.007128	3.328	191,176
Kostenki14	Ust_Ishim	Mbuti	-0.0075	0.00529	-1.423	1,069,412	-0.0066	0.005434	-1.21	1,069,393
Kostenki14	GoyetQ116	Mbuti	-0.0119	0.00542	-2.195	527,458	-0.0137	0.005551	-2.475	527,445
Kostenki14	Vestonice16	Mbuti	2.00E-04	0.005147	0.048	714,424	-0.0015	0.005361	-0.281	714,399
Kostenki14	Vestonice13_lc	Mbuti	0.0247	0.028038	0.88	4,265	-0.0134	0.029699	-0.45	4,264
Kostenki14	Vestonice43	Mbuti	0.0043	0.007067	0.61	122,853	0.0028	0.007213	0.393	122,850
Kostenki14	Vestonice14	Mbuti	-0.0099	0.006897	-1.432	111,419	-0.0112	0.007219	-1.557	111,416
Kostenki14	Vestonice15	Mbuti	-0.0139	0.011645	-1.194	28,523	-0.0153	0.012115	-1.265	28,521
Kostenki14	Villabruna	Mbuti	-0.0054	0.004854	-1.117	810,272	-0.0075	0.005026	-1.501	810,249
Kostenki14	Loschbour	Mbuti	-0.0142	0.004673	-3.043	1,002,322	-0.0207	0.004799	-4.31	1,002,307
Kostenki14	LaBrana	Mbuti	-0.0145	0.004616	-3.136	998,853	-0.0201	0.004812	-4.186	998,834
Kostenki14	ElMiron	Mbuti	-0.01	0.005127	-1.942	615,085	-0.0115	0.005147	-2.227	615,069
Kostenki14	Borsuka (C7/675)	Mbuti	-0.0037	0.010055	-0.371	39,872	-0.0102	0.010069	-1.011	39,870

Supplementary Table 9. Z-scores for D(Kostenki 14, X; Han/Dai, Mbuti), related to STAR Methods (Ancient DNA: *Nuclear DNA analysis*).

W	X	Y	Z	D	stderr	Zscore	nsnps
Ust_Ishim	Stuttgart	Han.DG	Mbuti.DG	0.0118	0.005172	2.284	1,102,364
Oase	Stuttgart	Han.DG	Mbuti.DG	-0.0163	0.006493	-2.518	188,418
Kostenki14	Stuttgart	Han.DG	Mbuti.DG	0.0059	0.004829	1.229	1,051,510
GoyetQ116	Stuttgart	Han.DG	Mbuti.DG	0.0186	0.005067	3.669	520,386
Vestonice16	Stuttgart	Han.DG	Mbuti.DG	0.0058	0.005167	1.12	704,244
Vestonice43	Stuttgart	Han.DG	Mbuti.DG	-0.0067	0.006829	-0.979	120,854
Vestonice14	Stuttgart	Han.DG	Mbuti.DG	0.0154	0.006317	2.439	109,621
Vestonice13_lc	Stuttgart	Han.DG	Mbuti.DG	0.0094	0.026282	0.359	4,197
Vestonice15	Stuttgart	Han.DG	Mbuti.DG	0.0098	0.010481	0.939	28,050
ElMiron	Stuttgart	Han.DG	Mbuti.DG	0.0157	0.004748	3.297	605,656
Villabruna	Stuttgart	Han.DG	Mbuti.DG	0.0126	0.004795	2.62	799,874
Loschbour	Stuttgart	Han.DG	Mbuti.DG	0.0272	0.004445	6.118	1,029,389
LaBrana	Stuttgart	Han.DG	Mbuti.DG	0.0255	0.004538	5.609	1,014,774
MA1	Stuttgart	Han.DG	Mbuti.DG	0.0313	0.004735	6.611	774,395
Borsuka (C7/675)	Stuttgart	Han.DG	Mbuti.DG	0.0043	0.008886	0.489	39,420

Supplementary Table 10. Z-scores for D(W, Stuttgart; Han, Mbuti), related to STAR Methods (Ancient DNA: *Nuclear DNA analysis*).

A	B	C	All SNPs				Transversions Only			
			f3	stderr	Zscore	nsnps	f3	stderr	Zscore	nsnps
Bacho Kiro 1653	Borsuka (C7/675)	Mbuti.DG	0.276651	0.006516	42.457	26,605	0.301444	0.013918	21.659	5,004
Vestonice16	Borsuka (C7/675)	Mbuti.DG	0.264994	0.007178	36.917	22,288	0.277205	0.014067	19.706	4,176
Sunghir3	Borsuka (C7/675)	Mbuti.DG	0.262109	0.006506	40.285	27,454	0.273746	0.013652	20.052	5,124
Villabruna	Borsuka (C7/675)	Mbuti.DG	0.264726	0.006639	39.873	24,499	0.272336	0.013198	20.634	4,543
Bichon	Borsuka (C7/675)	Mbuti.DG	0.267142	0.006525	40.939	27,496	0.272106	0.01267	21.476	5,129
GoyetQ116	Borsuka (C7/675)	Mbuti.DG	0.260682	0.007777	33.521	17,238	0.269117	0.016441	16.369	3,175
PesteraMuierii1	Borsuka (C7/675)	Mbuti.DG	0.268248	0.006468	41.474	27,555	0.268256	0.013487	19.891	5,153
Karelia	Borsuka (C7/675)	Mbuti.DG	0.258546	0.006304	41.012	26,650	0.264444	0.013514	19.568	4,949
Kostenki14	Borsuka (C7/675)	Mbuti.DG	0.262367	0.006693	39.2	27,355	0.261269	0.013205	19.785	5,124
Motala	Borsuka (C7/675)	Mbuti.DG	0.261298	0.005522	47.32	28,870	0.255957	0.011131	22.994	5,379
Loschbour	Borsuka (C7/675)	Mbuti.DG	0.267084	0.006207	43.032	27,108	0.251418	0.011891	21.144	5,058
ElMiron	Borsuka (C7/675)	Mbuti.DG	0.247546	0.007208	34.344	20,101	0.250693	0.014896	16.83	3,751
LaBrana	Borsuka (C7/675)	Mbuti.DG	0.262591	0.0065	40.4	26,541	0.250003	0.013174	18.977	4,967
MA1	Borsuka (C7/675)	Mbuti.DG	0.250837	0.007347	34.141	19,966	0.247571	0.015146	16.346	3,772
Kotias	Borsuka (C7/675)	Mbuti.DG	0.240631	0.006397	37.616	27,582	0.230743	0.012895	17.894	5,156
Bacho Kiro (CC7_335)	Borsuka (C7/675)	Mbuti.DG	0.220109	0.006799	32.372	22,054	0.228953	0.014123	16.211	4,130
Ust_Ishim	Borsuka (C7/675)	Mbuti.DG	0.220797	0.00644	34.283	27,589	0.227662	0.012641	18.009	5,165
Tianyuan	Borsuka (C7/675)	Mbuti.DG	0.220542	0.006415	34.38	25,228	0.217167	0.013294	16.336	4,745
Bacho Kiro (BB7_240)	Borsuka (C7/675)	Mbuti.DG	0.214505	0.006604	32.481	23,880	0.214027	0.013523	15.827	4,470
Oase	Borsuka (C7/675)	Mbuti.DG	0.215143	0.011063	19.447	6,697	0.197175	0.023243	8.483	1,389

Supplementary Table 11. *f3*-statistics for *f3*(A, Borsuka; Mbuti), related to Figure 6A.

W	X	Y	Z	All SNPs						Transversions Only					
				D	stderr	Zscore	BABA	ABBA	nsnps	D	stderr	Zscore	BABA	ABBA	nsnps
GoyetQ116	Vestonice16	Borsuka_C7_675	Mbuti.DG	-0.013	0.02023	-0.642	1,085	1,113	22,066	-0.0759	0.041563	-1.826	180	210	4,064
GoyetQ116	Kostenki14	Borsuka_C7_675	Mbuti.DG	-0.0084	0.018058	-0.465	1,309	1,331	25,092	-0.0201	0.037826	-0.532	233	243	4,590
GoyetQ116	LaBrana	Borsuka_C7_675	Mbuti.DG	-0.0043	0.01726	-0.248	1,285	1,296	24,409	0.0355	0.039384	0.902	231	215	4,465
GoyetQ116	ElMiron	Borsuka_C7_675	Mbuti.DG	0.0332	0.020134	1.649	1,015	950	20,384	0.0373	0.044625	0.835	191	177	3,777
GoyetQ116	Villabruna	Borsuka_C7_675	Mbuti.DG	-0.0088	0.018451	-0.475	1,243	1,265	23,624	-0.044	0.040724	-1.08	214	233	4,329
GoyetQ116	Tianyuan	Borsuka_C7_675	Mbuti.DG	0.0797	0.018092	4.407	1,447	1,233	24,086	0.0991	0.03986	2.486	264	216	4,426
GoyetQ116	Loschbour	Borsuka_C7_675	Mbuti.DG	-0.0181	0.017047	-1.062	1,248	1,294	24,070	0.0154	0.03701	0.416	220	214	4,396
GoyetQ116	Ust_Ishim	Borsuka_C7_675	Mbuti.DG	0.0887	0.017642	5.025	1,521	1,273	25,110	0.0698	0.037247	1.873	277	241	4,594
GoyetQ116	Satsurblia	Borsuka_C7_675	Mbuti.DG	0.0608	0.021433	2.836	841	745	14,423	0.0663	0.048907	1.356	162	142	2,643
GoyetQ116	Sunghir3	Borsuka_C7_675	Mbuti.DG	-0.0041	0.017083	-0.239	1,330	1,341	25,075	-0.0439	0.041	-1.071	217	236	4,589
GoyetQ116	BK_1653	Borsuka_C7_675	Mbuti.DG	-0.0397	0.017899	-2.219	1,232	1,334	24,746	-0.1182	0.039943	-2.959	199	252	4,548
GoyetQ116	MA1	Borsuka_C7_675	Mbuti.DG	0.0369	0.01958	1.884	1,036	962	18,434	0.0609	0.043808	1.39	204	180	3,400
GoyetQ116	BK_BB7_240	Borsuka_C7_675	Mbuti.DG	0.107	0.017664	6.06	1,396	1,126	22,841	0.1246	0.040853	3.049	253	197	4,212
GoyetQ116	BK_CC7_335	Borsuka_C7_675	Mbuti.DG	0.0938	0.018799	4.99	1,313	1,088	21,315	0.0638	0.041899	1.523	222	196	3,927
GoyetQ116	PesteraMuierii	Borsuka_C7_675	Mbuti.DG	-0.0325	0.018234	-1.782	1,277	1,363	25,099	-0.026	0.039937	-0.65	231	243	4,593
Vestonice16	GoyetQ116	Borsuka_C7_675	Mbuti.DG	0.013	0.02023	0.642	1,113	1,085	22,066	0.0759	0.041563	1.826	210	180	4,064
Vestonice16	Kostenki14	Borsuka_C7_675	Mbuti.DG	0.0275	0.015943	1.724	1,729	1,636	32,500	0.0397	0.033697	1.179	326	301	6,042
Vestonice16	LaBrana	Borsuka_C7_675	Mbuti.DG	0.0162	0.015899	1.02	1,698	1,644	31,545	0.0683	0.033899	2.016	326	284	5,876
Vestonice16	ElMiron	Borsuka_C7_675	Mbuti.DG	0.0264	0.017804	1.485	1,360	1,290	26,244	0.0386	0.037331	1.035	258	239	4,907
Vestonice16	Villabruna	Borsuka_C7_675	Mbuti.DG	0.0086	0.015624	0.548	1,607	1,580	30,590	-0.0122	0.035359	-0.346	285	292	5,682
Vestonice16	Tianyuan	Borsuka_C7_675	Mbuti.DG	0.096	0.015494	6.193	1,879	1,550	31,227	0.1344	0.032372	4.152	357	272	5,840
Vestonice16	Loschbour	Borsuka_C7_675	Mbuti.DG	0.0016	0.014784	0.112	1,631	1,625	31,082	0.0611	0.030169	2.024	319	283	5,754
Vestonice16	Ust_Ishim	Borsuka_C7_675	Mbuti.DG	0.1051	0.015676	6.708	1,936	1,568	32,515	0.1061	0.031917	3.323	368	297	6,046
Vestonice16	Satsurblia	Borsuka_C7_675	Mbuti.DG	0.0773	0.019676	3.928	1,081	926	18,771	0.1131	0.042722	2.648	215	172	3,513
Vestonice16	Sunghir3	Borsuka_C7_675	Mbuti.DG	0.0122	0.015672	0.78	1,667	1,627	32,476	-0.0052	0.035447	-0.145	292	295	6,040
Vestonice16	BK_1653	Borsuka_C7_675	Mbuti.DG	-0.0227	0.01611	-1.411	1,592	1,666	32,043	-0.0559	0.033882	-1.65	277	310	5,985
Vestonice16	MA1	Borsuka_C7_675	Mbuti.DG	0.0409	0.016801	2.433	1,298	1,196	23,870	0.0638	0.037892	1.684	252	222	4,468
Vestonice16	BK_BB7_240	Borsuka_C7_675	Mbuti.DG	0.107	0.016374	6.536	1,791	1,445	29,224	0.1256	0.033392	3.761	336	261	5,472
Vestonice16	BK_CC7_335	Borsuka_C7_675	Mbuti.DG	0.106	0.016444	6.445	1,690	1,366	27,284	0.1091	0.035853	3.042	302	242	5,102
Vestonice16	PesteraMuierii	Borsuka_C7_675	Mbuti.DG	0.009	0.015784	0.571	1,688	1,657	32,500	0.0225	0.034302	0.656	309	295	6,045
Kostenki14	GoyetQ116	Borsuka_C7_675	Mbuti.DG	0.0084	0.018058	0.465	1,331	1,309	25,092	0.0201	0.037826	0.532	243	233	4,590
Kostenki14	Vestonice16	Borsuka_C7_675	Mbuti.DG	-0.0275	0.015943	-1.724	1,636	1,729	32,500	-0.0397	0.033697	-1.179	301	326	6,042
Kostenki14	LaBrana	Borsuka_C7_675	Mbuti.DG	-0.0021	0.01391	-0.15	2,071	2,080	38,372	0.0317	0.03122	1.016	395	371	7,153
Kostenki14	ElMiron	Borsuka_C7_675	Mbuti.DG	0.0108	0.016099	0.671	1,552	1,519	29,335	0.0166	0.034504	0.482	311	301	5,482
Kostenki14	Villabruna	Borsuka_C7_675	Mbuti.DG	-0.0143	0.014921	-0.957	1,883	1,938	35,537	-0.032	0.030113	-1.061	343	365	6,585
Kostenki14	Tianyuan	Borsuka_C7_675	Mbuti.DG	0.0886	0.01453	6.1	2,203	1,844	36,716	0.1035	0.030803	3.36	425	345	6,863
Kostenki14	Loschbour	Borsuka_C7_675	Mbuti.DG	-0.0084	0.012744	-0.657	2,007	2,041	37,950	0.0214	0.026585	0.805	387	371	7,043
Kostenki14	Ust_Ishim	Borsuka_C7_675	Mbuti.DG	0.0881	0.014059	6.268	2,393	2,006	39,869	0.069	0.028624	2.412	439	382	7,425
Kostenki14	Satsurblia	Borsuka_C7_675	Mbuti.DG	0.0597	0.017168	3.475	1,324	1,175	23,112	0.0706	0.038408	1.839	263	228	4,331
Kostenki14	Sunghir3	Borsuka_C7_675	Mbuti.DG	-0.001	0.013978	-0.072	2,033	2,037	39,816	-0.0337	0.031219	-1.079	358	383	7,417
Kostenki14	BK_1653	Borsuka_C7_675	Mbuti.DG	-0.0333	0.014092	-2.363	1,983	2,120	38,603	-0.0937	0.030342	-3.089	342	413	7,223
Kostenki14	MA1	Borsuka_C7_675	Mbuti.DG	0.0261	0.016244	1.609	1,547	1,468	28,991	0.0383	0.035248	1.088	305	282	5,436
Kostenki14	BK_BB7_240	Borsuka_C7_675	Mbuti.DG	0.0976	0.014301	6.823	2,137	1,757	34,593	0.1004	0.031756	3.163	392	321	6,491
Kostenki14	BK_CC7_335	Borsuka_C7_675	Mbuti.DG	0.0942	0.015581	6.046	1,975	1,635	32,091	0.0824	0.033831	2.436	362	307	6,016
Kostenki14	PesteraMuierii	Borsuka_C7_675	Mbuti.DG	-0.0144	0.013453	-1.07	2,077	2,138	39,851	-0.0186	0.029497	-0.63	387	402	7,425
LaBrana	GoyetQ116	Borsuka_C7_675	Mbuti.DG	0.0043	0.01726	0.248	1,296	1,285	24,409	-0.0355	0.039384	-0.902	215	231	4,465
LaBrana	Vestonice16	Borsuka_C7_675	Mbuti.DG	-0.0162	0.015899	-1.02	1,644	1,698	31,545	-0.0683	0.033899	-2.016	284	326	5,876
LaBrana	Kostenki14	Borsuka_C7_675	Mbuti.DG	0.0021	0.01391	0.15	2,080	2,071	38,372	-0.0317	0.03122	-1.016	371	395	7,153
LaBrana	ElMiron	Borsuka_C7_675	Mbuti.DG	0.022	0.016671	1.318	1,391	1,331	28,558	0.006	0.038105	0.158	260	257	5,336
LaBrana	Villabruna	Borsuka_C7_675	Mbuti.DG	-0.0102	0.015851	-0.646	1,594	1,627	34,493	-0.0662	0.033347	-1.985	286	327	6,385
LaBrana	Tianyuan	Borsuka_C7_675	Mbuti.DG	0.0846	0.014798	5.715	2,111	1,781	35,611	0.0756	0.031885	2.37	392	337	6,660
LaBrana	Loschbour	Borsuka_C7_675	Mbuti.DG	-0.0092	0.013849	-0.661	1,678	1,709	36,706	-0.0058	0.030945	-0.186	306	310	6,822
LaBrana	Ust_Ishim	Borsuka_C7_675	Mbuti.DG	0.0904	0.014012	6.448	2,334	1,947	38,542	0.0482	0.029927	1.611	414	376	7,193
LaBrana	Satsurblia	Borsuka_C7_675	Mbuti.DG	0.0611	0.017854	3.422	1,269	1,123	22,324	0.0709	0.04008	1.768	240	208	4,202
LaBrana	Sunghir3	Borsuka_C7_675	Mbuti.DG	0.0033	0.013742	0.24	2,035	2,022	38,491	-0.0559	0.030687	-1.822	346	387	7,182
LaBrana	BK_1653	Borsuka_C7_675	Mbuti.DG	-0.0345	0.014504	-2.382	1,893	2,029	37,308	-0.1256	0.031756	-3.954	319	410	6,987
LaBrana	MA1	Borsuka_C7_675	Mbuti.DG	0.0325	0.016074	2.024	1,541	1,444	28,092	-0.0085	0.036495	-0.232	279	283	5,265
LaBrana	BK_BB7_240	Borsuka_C7_675	Mbuti.DG	0.0913	0.015045	6.066	2,075	1,728	33,506	0.0743	0.032662	2.274	371	320	6,292
LaBrana	BK_CC7_335	Borsuka_C7_675	Mbuti.DG	0.092	0.015356	5.992	1,916	1,593	31,086	0.0412	0.033307	1.237	328	302	5,838
LaBrana	PesteraMuierii	Borsuka_C7_675	Mbuti.DG	-0.0145	0.013965	-1.039	2,003	2,062	38,523	-0.0559	0.03071	-1.819	353	395	7,191
ElMiron	GoyetQ116	Borsuka_C7_675	Mbuti.DG	-0.0332	0.020134	-1.649	950	1,015	20,384	-0.0373	0.044625	-0.835	177	191	3,777
ElMiron	Vestonice16	Borsuka_C7_675	Mbuti.DG	-0.0264	0.017804	-1.485	1,290	1,360	26,244	-0.0386	0.037331	-1.035	239	258	4,907
ElMiron	Kostenki14	Borsuka_C7_675	Mbuti.DG	-0.0108	0.016099	-0.671	1,519	1,552	29,335	-0.0166	0.034504	-0.482	301	311	5,482
ElMiron	LaBrana	Borsuka_C7_675	Mbuti.DG	-0.022	0.016671	-1.318	1,331	1,391	28,558	-0.006	0.038105	-0.158	257	260	5,336
ElMiron	Villabruna	Borsuka_C7_675	Mbuti.DG	-0.0311	0.016988	-1.831	1,325	1,410	27,942	-0.0677	0.038075	-1.777	230	263	5,203
ElMiron	Tianyuan	Borsuka_C7_675	Mbuti.DG	0.0667	0.016788	3.973	1,614	1,412	28,420	0.081	0.035271	2.297	307	261	5,328
ElMiron	Loschbour	Borsuka_C7_675	Mbuti.DG	-0.0302	0.01557	-1.939	1,301	1,382	28,080	-0.0013	0.034891	-0.036	251	251	5,237
ElMiron	Ust_Ishim	Borsuka_C7_675	Mbuti.DG	0.0648	0.016238	3.99	1,697	1,491	29,345	0.0485	0.035215	1.378	326	296	5,484
ElMiron	Satsurblia	Borsuka_C7_675	Mbuti.DG	0.0396	0.020784	1.903	941	870	16,834	0.0641	0.045385	1.411	189	166	3,161
ElMiron	Sunghir3	Borsuka_C7_675	Mbuti.DG	-0.0177	0.016088	-1.098	1,528	1,583	29,308	-0.0644	0.034516	-1.865	276	314	5,479
ElMiron	BK_1653	Borsuka_C7_675	Mbuti.DG	-0.0677	0.016637	-4.068	1,378	1,578	29,012	-0.1352	0.036441	-3.711	238	313	5,430
ElMiron	MA1	Borsuka_C7_675	Mbuti.DG	0.0169	0.018858	0.897	1,156	1,118	21,562	0.0028	0.039934	0.071	239	237	4,088
ElMiron	BK_BB7_240	Borsuka_C7_675	Mbuti.DG	0.0797	0.016429	4.849	1,581	1,347	26,597	0.0766	0.036354	2.106	296	254	5,014
ElMiron	BK_CC7_335	Borsuka_C7_675	Mbuti.DG	0.0639	0.01722	3.708	1,470	1,293	24,842	0.028	0.038188	0.732	260	246	4,671
ElMiron	PesteraMuierii	Borsuka_C7_675	Mbuti.DG	-0.0316	0.015727	-2.008	1,470	1,566	29,331	-0.0532	0.035072	-1.515	280	312	5,483
Villabruna	GoyetQ116	Borsuka_C7_675	Mbuti.DG	0.0088	0.018451	0.475	1,265	1,243	23,624	0.044	0.040724	1.08	233	214	4,329
Villabruna	Vestonice16	Borsuka_C7_675	Mbuti.DG	-0.0086	0.015624	-0.548	1,580	1,607	30,590	0.0122	0.035359	0.346	292	285	5,682
Villabruna	Kostenki14	Borsuka_C7_675	Mbuti.DG	0.0143	0.014921	0.957	1,938	1,883	35,537	0.032	0.030113	1.061	365	343	6,585
Villabruna	LaBrana	Borsuka_C7_675	Mbuti.DG	0.0102	0.015851	0.646	1,627	1,594	34,493	0.0662	0.033347	1.985	327	286	6,385
Villabruna	ElMiron	Borsuka_C7_675	Mbuti.DG	0.0311	0.016988	1.831	1,410	1,325	27,942	0.0677	0.038075	1.777	263	230	5,203
Villabruna	Tianyuan	Borsuka_C7_675	Mbuti.DG	0.0911	0.014705	6.198	2,050	1,707	33,865	0.1228	0.031234	3.932	407	318	6,295
Villabruna	Loschbour	Borsuka_C7_675	Mbuti.DG	0.0011	0.014907	0.075	1,496	1,493	33,954	0.0683	0.031265	2.184	301	263	6,270
Villabruna	Ust_Ishim	Borsuka_C7_675	Mbuti.DG	0.0994	0.014318	6.94	2,169	1,777	35,567	0.0935	0.02971	3.148	409	339	6,591
Villabruna	Satsurblia	Borsuka_C7_675	Mbuti.DG	0.049	0.018993	2.578	1,113	1,009	20,489	0.0738	0.040919	1.804	217	187	3,810
Villabruna	Sunghir3	Borsuka_C7_675	Mbuti.DG	0.0137	0.014857	0.921	1,871	1,820	35,521	-0.0026	0.032568	-0.078	336	338	6,582
Villabruna	BK_1653	Borsuka_C7_675	Mbuti.DG	-0.0233	0.015048	-1.546	1,785	1,870	34,958	-0.0612	0.032272	-1.896	311	352	6,501
Villabruna	MA1	Borsuka_C7_675	Mbuti.DG	0.0388	0.016359	2.372	1,440	1,333	26,048	0.0862	0.035305	2.442	289	243	4,877
Villabruna	BK_BB7_240	Borsuka_C7_675	Mbuti.DG	0.109	0.015181	7.177	1,978	1,589	31,728	0.1331	0.03285	4.05	377	289	5,923
Villabruna	BK_CC7_335	Borsuka_C7_675	Mbuti.DG	0.1022	0.015853	6.448	1,831	1,491	29,621	0.1111	0.033283	3.338	328	263	5,512
Villabruna	PesteraMuierii	Borsuka_C7_675	Mbuti.DG	0.0036	0.014208	0.254	1,906	1,893	35,551	0.0178	0.032415	0.549	349	337	6,590

Supplementary Table 12. *D*-statistics for *D*(W,X; Borsuka, Mbuti), related to STAR Methods (Ancient DNA: *Nuclear DNA analysis*).

W	X	Y	Z	All SNPs						Transversions Only					
				D	stderr	Zscore	BABA	ABBA	nsnps	D	stderr	Zscore	BABA	ABBA	nsnps
Tianyuan	GoyetQ116	Borsuka_C7_675	Mbuti.DG	-0.0797	0.018092	-4.407	1,233	1,447	24,086	-0.0991	0.03986	-2.486	216	264	4,426
Tianyuan	Vestonice16	Borsuka_C7_675	Mbuti.DG	-0.096	0.015494	-6.193	1,550	1,879	31,227	-0.1344	0.032372	-4.152	272	357	5,840
Tianyuan	Kostenki14	Borsuka_C7_675	Mbuti.DG	-0.0886	0.01453	-6.1	1,844	2,203	36,716	-0.1035	0.030803	-3.36	345	425	6,863
Tianyuan	LaBrana	Borsuka_C7_675	Mbuti.DG	-0.0846	0.014798	-5.715	1,781	2,111	35,611	-0.0756	0.031885	-2.37	337	392	6,660
Tianyuan	ElMiron	Borsuka_C7_675	Mbuti.DG	-0.0667	0.016788	-3.973	1,412	1,614	28,420	-0.081	0.035271	-2.297	261	307	5,328
Tianyuan	Villabruna	Borsuka_C7_675	Mbuti.DG	-0.0911	0.014705	-6.198	1,707	2,050	33,865	-0.1228	0.031234	-3.932	318	407	6,295
Tianyuan	Loschbour	Borsuka_C7_675	Mbuti.DG	-0.0925	0.013137	-7.039	1,781	2,144	35,091	-0.0687	0.029648	-2.316	338	388	6,540
Tianyuan	Ust_Ishim	Borsuka_C7_675	Mbuti.DG	0.0065	0.014746	0.441	1,976	1,951	36,753	-0.0233	0.031808	-0.733	347	364	6,871
Tianyuan	Satsurblia	Borsuka_C7_675	Mbuti.DG	-0.0223	0.017683	-1.263	1,125	1,176	21,251	-0.0021	0.040714	-0.051	221	222	3,990
Tianyuan	Sunghir3	Borsuka_C7_675	Mbuti.DG	-0.0907	0.01397	-6.49	1,817	2,179	36,704	-0.1429	0.031806	-4.491	307	410	6,864
Tianyuan	BK_1653	Borsuka_C7_675	Mbuti.DG	-0.1168	0.014467	-8.077	1,801	2,277	36,013	-0.1895	0.029711	-6.38	308	452	6,772
Tianyuan	MA1	Borsuka_C7_675	Mbuti.DG	-0.0586	0.016011	-3.659	1,374	1,545	26,920	-0.0686	0.036194	-1.896	268	307	5,066
Tianyuan	BK_BB7_240	Borsuka_C7_675	Mbuti.DG	0.0109	0.01537	0.707	1,782	1,744	32,524	-0.0031	0.033911	-0.091	332	334	6,130
Tianyuan	BK_CC7_335	Borsuka_C7_675	Mbuti.DG	0.0099	0.015043	0.657	1,658	1,626	30,324	-0.0097	0.034508	-0.281	289	294	5,700
Tianyuan	PesteraMuierii	Borsuka_C7_675	Mbuti.DG	-0.0989	0.013708	-7.216	1,846	2,251	36,735	-0.116	0.030746	-3.772	346	436	6,870
Loschbour	GoyetQ116	Borsuka_C7_675	Mbuti.DG	0.0181	0.017047	1.062	1,294	1,248	24,070	-0.0154	0.03701	-0.416	214	220	4,396
Loschbour	Vestonice16	Borsuka_C7_675	Mbuti.DG	-0.0016	0.014784	-0.112	1,625	1,631	31,082	-0.0611	0.030169	-2.024	283	319	5,754
Loschbour	Kostenki14	Borsuka_C7_675	Mbuti.DG	0.0084	0.012744	0.657	2,041	2,007	37,950	-0.0214	0.026585	-0.805	371	387	7,043
Loschbour	LaBrana	Borsuka_C7_675	Mbuti.DG	0.0092	0.013849	0.661	1,709	1,678	36,706	0.0058	0.030945	0.186	310	306	6,822
Loschbour	ElMiron	Borsuka_C7_675	Mbuti.DG	0.0302	0.01557	1.939	1,382	1,301	28,080	0.0013	0.034891	0.036	251	251	5,237
Loschbour	Villabruna	Borsuka_C7_675	Mbuti.DG	-0.0011	0.014907	-0.075	1,493	1,496	33,954	-0.0683	0.031265	-2.184	263	301	6,270
Loschbour	Tianyuan	Borsuka_C7_675	Mbuti.DG	0.0925	0.013137	7.039	2,144	1,781	35,091	0.0687	0.029648	2.316	388	338	6,540
Loschbour	Ust_Ishim	Borsuka_C7_675	Mbuti.DG	0.0982	0.012908	7.607	2,372	1,948	38,152	0.0467	0.026906	1.737	421	384	7,091
Loschbour	Satsurblia	Borsuka_C7_675	Mbuti.DG	0.073	0.016204	4.505	1,249	1,079	22,120	0.0617	0.036561	1.687	234	207	4,140
Loschbour	Sunghir3	Borsuka_C7_675	Mbuti.DG	0.0104	0.012765	0.812	2,048	2,006	38,099	-0.0568	0.027502	-2.065	350	392	7,079
Loschbour	BK_1653	Borsuka_C7_675	Mbuti.DG	-0.0254	0.013558	-1.872	1,892	1,991	36,887	-0.1173	0.029228	-4.012	320	405	6,890
Loschbour	MA1	Borsuka_C7_675	Mbuti.DG	0.0457	0.015038	3.041	1,557	1,420	27,730	0.0128	0.033917	0.378	295	288	5,176
Loschbour	BK_BB7_240	Borsuka_C7_675	Mbuti.DG	0.107	0.013741	7.785	2,082	1,680	33,067	0.0834	0.028899	2.886	386	327	6,196
Loschbour	BK_CC7_335	Borsuka_C7_675	Mbuti.DG	0.0927	0.014186	6.534	1,897	1,575	30,625	0.0309	0.030893	0.999	321	302	5,724
Loschbour	PesteraMuierii	Borsuka_C7_675	Mbuti.DG	-0.0083	0.012766	-0.65	2,002	2,036	38,133	-0.0453	0.027691	-1.637	353	386	7,088
Ust_Ishim	GoyetQ116	Borsuka_C7_675	Mbuti.DG	-0.0887	0.017642	-5.025	1,273	1,521	25,110	-0.0698	0.037247	-1.873	241	277	4,594
Ust_Ishim	Vestonice16	Borsuka_C7_675	Mbuti.DG	-0.1051	0.015676	-6.708	1,568	1,936	32,515	-0.1061	0.031917	-3.323	297	368	6,046
Ust_Ishim	Kostenki14	Borsuka_C7_675	Mbuti.DG	-0.0881	0.014059	-6.268	2,006	2,393	39,869	-0.069	0.028624	-2.412	382	439	7,425
Ust_Ishim	LaBrana	Borsuka_C7_675	Mbuti.DG	-0.0904	0.014012	-6.448	1,947	2,334	38,542	-0.0482	0.029927	-1.611	376	414	7,193
Ust_Ishim	ElMiron	Borsuka_C7_675	Mbuti.DG	-0.0648	0.016238	-3.99	1,491	1,697	29,345	-0.0485	0.035215	-1.378	296	326	5,484
Ust_Ishim	Villabruna	Borsuka_C7_675	Mbuti.DG	-0.0994	0.014318	-6.94	1,777	2,169	35,567	-0.0935	0.02971	-3.148	339	409	6,591
Ust_Ishim	Tianyuan	Borsuka_C7_675	Mbuti.DG	-0.0065	0.014746	-0.441	1,951	1,976	36,753	0.0233	0.031808	0.733	364	347	6,871
Ust_Ishim	Loschbour	Borsuka_C7_675	Mbuti.DG	-0.0982	0.012908	-7.607	1,948	2,372	38,152	-0.0467	0.026906	-1.737	384	421	7,091
Ust_Ishim	Satsurblia	Borsuka_C7_675	Mbuti.DG	-0.0125	0.017924	-0.697	1,267	1,299	23,250	-0.0099	0.039705	-0.25	235	240	4,363
Ust_Ishim	Sunghir3	Borsuka_C7_675	Mbuti.DG	-0.0861	0.013711	-6.279	2,081	2,473	40,049	-0.1011	0.030041	-3.366	371	455	7,470
Ust_Ishim	BK_1653	Borsuka_C7_675	Mbuti.DG	-0.1177	0.014057	-8.375	1,924	2,437	38,697	-0.1541	0.028834	-5.345	351	479	7,245
Ust_Ishim	MA1	Borsuka_C7_675	Mbuti.DG	-0.0596	0.015757	-3.784	1,470	1,656	29,132	-0.0558	0.034061	-1.639	279	312	5,469
Ust_Ishim	BK_BB7_240	Borsuka_C7_675	Mbuti.DG	0.0092	0.014605	0.631	1,930	1,895	34,671	0.0232	0.03164	0.734	366	350	6,510
Ust_Ishim	BK_CC7_335	Borsuka_C7_675	Mbuti.DG	0.0047	0.015257	0.31	1,758	1,741	32,142	-0.0134	0.034388	-0.389	316	325	6,026
Ust_Ishim	PesteraMuierii	Borsuka_C7_675	Mbuti.DG	-0.102	0.014016	-7.279	1,988	2,439	40,085	-0.0879	0.029821	-2.949	382	455	7,479
Satsurblia	GoyetQ116	Borsuka_C7_675	Mbuti.DG	-0.0608	0.021433	-2.836	745	841	14,423	-0.0663	0.048907	-1.356	142	162	2,643
Satsurblia	Vestonice16	Borsuka_C7_675	Mbuti.DG	-0.0773	0.019676	-3.928	926	1,081	18,771	-0.1131	0.042722	-2.648	172	215	3,513
Satsurblia	Kostenki14	Borsuka_C7_675	Mbuti.DG	-0.0597	0.017168	-3.475	1,175	1,324	23,112	-0.0706	0.038408	-1.839	228	263	4,331
Satsurblia	LaBrana	Borsuka_C7_675	Mbuti.DG	-0.0611	0.017854	-3.422	1,123	1,269	22,324	-0.0709	0.04008	-1.768	208	240	4,202
Satsurblia	ElMiron	Borsuka_C7_675	Mbuti.DG	-0.0396	0.020784	-1.903	870	941	16,834	-0.0641	0.045385	-1.411	166	189	3,161
Satsurblia	Villabruna	Borsuka_C7_675	Mbuti.DG	-0.049	0.018993	-2.578	1,009	1,113	20,489	-0.0738	0.040919	-1.804	187	217	3,810
Satsurblia	Tianyuan	Borsuka_C7_675	Mbuti.DG	0.0223	0.017683	1.263	1,176	1,125	21,251	0.0021	0.040714	0.051	222	221	3,990
Satsurblia	Loschbour	Borsuka_C7_675	Mbuti.DG	-0.073	0.016204	-4.505	1,079	1,249	22,120	-0.0617	0.036561	-1.687	207	234	4,140
Satsurblia	Ust_Ishim	Borsuka_C7_675	Mbuti.DG	0.0125	0.017924	0.697	1,299	1,267	23,250	0.0099	0.039705	0.25	240	235	4,363
Satsurblia	Sunghir3	Borsuka_C7_675	Mbuti.DG	-0.0512	0.016516	-3.102	1,172	1,299	23,225	-0.1	0.041605	-2.404	201	245	4,360
Satsurblia	BK_1653	Borsuka_C7_675	Mbuti.DG	-0.0854	0.0173	-4.937	1,127	1,337	22,428	-0.1813	0.039593	-4.579	193	279	4,224
Satsurblia	MA1	Borsuka_C7_675	Mbuti.DG	-0.0381	0.020737	-1.836	856	923	16,908	-0.07	0.043921	-1.593	162	186	3,176
Satsurblia	BK_BB7_240	Borsuka_C7_675	Mbuti.DG	0.032	0.017939	1.785	1,171	1,098	20,095	0.0528	0.038937	1.357	227	204	3,779
Satsurblia	BK_CC7_335	Borsuka_C7_675	Mbuti.DG	0.0363	0.018857	1.925	1,048	974	18,655	-0.0166	0.044464	-0.374	185	191	3,525
Satsurblia	PesteraMuierii	Borsuka_C7_675	Mbuti.DG	-0.0567	0.017798	-3.187	1,196	1,340	23,247	-0.099	0.038942	-2.541	214	261	4,363
Sunghir3	GoyetQ116	Borsuka_C7_675	Mbuti.DG	0.0041	0.017083	0.239	1,341	1,330	25,075	0.0439	0.041	1.071	236	217	4,589
Sunghir3	Vestonice16	Borsuka_C7_675	Mbuti.DG	-0.0122	0.015672	-0.78	1,627	1,667	32,476	0.0052	0.035447	0.145	295	292	6,040
Sunghir3	Kostenki14	Borsuka_C7_675	Mbuti.DG	0.001	0.013978	0.072	2,037	2,033	39,816	0.0337	0.031219	1.079	383	358	7,417
Sunghir3	LaBrana	Borsuka_C7_675	Mbuti.DG	-0.0033	0.013742	-0.24	2,022	2,035	38,491	0.0559	0.030687	1.822	387	346	7,182
Sunghir3	ElMiron	Borsuka_C7_675	Mbuti.DG	0.0177	0.016088	1.098	1,583	1,528	29,308	0.0644	0.034516	1.865	314	276	5,479
Sunghir3	Villabruna	Borsuka_C7_675	Mbuti.DG	-0.0137	0.014857	-0.921	1,820	1,871	35,521	0.0026	0.032568	0.078	338	336	6,582
Sunghir3	Tianyuan	Borsuka_C7_675	Mbuti.DG	0.0907	0.01397	6.49	2,179	1,817	36,704	0.1429	0.031806	4.491	410	307	6,864
Sunghir3	Loschbour	Borsuka_C7_675	Mbuti.DG	-0.0104	0.012765	-0.812	2,006	2,048	38,099	0.0568	0.027502	2.065	392	350	7,079
Sunghir3	Ust_Ishim	Borsuka_C7_675	Mbuti.DG	0.0861	0.013711	6.279	2,473	2,081	40,049	0.1011	0.030041	3.366	455	371	7,470
Sunghir3	Satsurblia	Borsuka_C7_675	Mbuti.DG	0.0512	0.016516	3.102	1,299	1,172	23,225	0.1	0.041605	2.404	245	201	4,360
Sunghir3	BK_1653	Borsuka_C7_675	Mbuti.DG	-0.0353	0.014333	-2.46	1,960	2,103	38,651	-0.0659	0.032258	-2.043	350	399	7,236
Sunghir3	MA1	Borsuka_C7_675	Mbuti.DG	0.0274	0.014928	1.834	1,603	1,518	29,100	0.0723	0.034972	2.067	306	265	5,464
Sunghir3	BK_BB7_240	Borsuka_C7_675	Mbuti.DG	0.0933	0.01394	6.69	2,174	1,803	34,632	0.1244	0.031186	3.988	419	326	6,505
Sunghir3	BK_CC7_335	Borsuka_C7_675	Mbuti.DG	0.0882	0.014375	6.135	1,963	1,645	32,099	0.1238	0.033923	3.648	362	282	6,016
Sunghir3	PesteraMuierii	Borsuka_C7_675	Mbuti.DG	-0.0145	0.013547	-1.074	2,074	2,135	40,034	0.0137	0.031931	0.43	377	367	7,470

Supplementary Table 12 continued. *D*-statistics for *D*(W,X; Borsuka, Mbuti), related to STAR Methods (Ancient DNA: *Nuclear DNA analysis*).

.

W	X	Y	Z	All SNPs						Transversions Only					
				D	stderr	Zscore	BABA	ABBA	nsnps	D	stderr	Zscore	BABA	ABBA	nsnps
BK_1653	GoyetQ116	Borsuka_C7_675	Mbuti.DG	0.0397	0.017899	2.219	1,334	1,232	24,746	0.1182	0.039943	2.959	252	199	4,548
BK_1653	Vestonice16	Borsuka_C7_675	Mbuti.DG	0.0227	0.01611	1.411	1,666	1,592	32,043	0.0559	0.033882	1.65	310	277	5,985
BK_1653	Kostenki14	Borsuka_C7_675	Mbuti.DG	0.0333	0.014092	2.363	2,120	1,983	38,603	0.0937	0.030342	3.089	413	342	7,223
BK_1653	LaBrana	Borsuka_C7_675	Mbuti.DG	0.0345	0.014504	2.382	2,029	1,893	37,308	0.1256	0.031756	3.954	410	319	6,987
BK_1653	ElMiron	Borsuka_C7_675	Mbuti.DG	0.0677	0.016637	4.068	1,578	1,378	29,012	0.1352	0.036441	3.711	313	238	5,430
BK_1653	Villabruna	Borsuka_C7_675	Mbuti.DG	0.0233	0.015048	1.546	1,870	1,785	34,958	0.0612	0.032272	1.896	352	311	6,501
BK_1653	Tianyuan	Borsuka_C7_675	Mbuti.DG	0.1168	0.014467	8.077	2,277	1,801	36,013	0.1895	0.029711	6.38	452	308	6,772
BK_1653	Loschbour	Borsuka_C7_675	Mbuti.DG	0.0254	0.013558	1.872	1,991	1,892	36,887	0.1173	0.029228	4.012	405	320	6,890
BK_1653	Ust_Ishim	Borsuka_C7_675	Mbuti.DG	0.1177	0.014057	8.375	2,437	1,924	38,697	0.1541	0.028834	5.345	479	351	7,245
BK_1653	Satsurblia	Borsuka_C7_675	Mbuti.DG	0.0854	0.0173	4.937	1,337	1,127	22,428	0.1813	0.039593	4.579	279	193	4,224
BK_1653	Sunghir3	Borsuka_C7_675	Mbuti.DG	0.0353	0.014333	2.46	2,103	1,960	38,651	0.0659	0.032258	2.043	399	350	7,236
BK_1653	MA1	Borsuka_C7_675	Mbuti.DG	0.0671	0.015907	4.22	1,635	1,429	28,207	0.1382	0.034188	4.042	337	255	5,305
BK_1653	BK_BB7_240	Borsuka_C7_675	Mbuti.DG	0.1303	0.014383	9.061	2,169	1,669	33,919	0.1907	0.030545	6.244	433	294	6,399
BK_1653	BK_CC7_335	Borsuka_C7_675	Mbuti.DG	0.1214	0.015097	8.043	2,035	1,594	31,484	0.1657	0.033384	4.963	401	287	5,934
BK_1653	PesteraMuierii	Borsuka_C7_675	Mbuti.DG	0.0223	0.013999	1.59	2,008	1,920	38,680	0.0879	0.032171	2.731	381	319	7,244
MA1	GoyetQ116	Borsuka_C7_675	Mbuti.DG	-0.0369	0.01958	-1.884	962	1,036	18,434	-0.0609	0.043808	-1.39	180	204	3,400
MA1	Vestonice16	Borsuka_C7_675	Mbuti.DG	-0.0409	0.016801	-2.433	1,196	1,298	23,870	-0.0638	0.037892	-1.684	222	252	4,468
MA1	Kostenki14	Borsuka_C7_675	Mbuti.DG	-0.0261	0.016244	-1.609	1,468	1,547	28,991	-0.0383	0.035248	-1.088	282	305	5,436
MA1	LaBrana	Borsuka_C7_675	Mbuti.DG	-0.0325	0.016074	-2.024	1,444	1,541	28,092	0.0085	0.036495	0.232	283	279	5,265
MA1	ElMiron	Borsuka_C7_675	Mbuti.DG	-0.0169	0.018858	-0.897	1,118	1,156	21,562	-0.0028	0.039934	-0.071	237	239	4,088
MA1	Villabruna	Borsuka_C7_675	Mbuti.DG	-0.0388	0.016359	-2.372	1,333	1,440	26,048	-0.0862	0.035305	-2.442	243	289	4,877
MA1	Tianyuan	Borsuka_C7_675	Mbuti.DG	0.0586	0.016011	3.659	1,545	1,374	26,920	0.0686	0.036194	1.896	307	268	5,066
MA1	Loschbour	Borsuka_C7_675	Mbuti.DG	-0.0457	0.015038	-3.041	1,420	1,557	27,730	-0.0128	0.033917	-0.378	288	295	5,176
MA1	Ust_Ishim	Borsuka_C7_675	Mbuti.DG	0.0596	0.015757	3.784	1,656	1,470	29,132	0.0558	0.034061	1.639	312	279	5,469
MA1	Satsurblia	Borsuka_C7_675	Mbuti.DG	0.0381	0.020737	1.836	923	856	16,908	0.07	0.043921	1.593	186	162	3,176
MA1	Sunghir3	Borsuka_C7_675	Mbuti.DG	-0.0274	0.014928	-1.834	1,518	1,603	29,100	-0.0723	0.034972	-2.067	265	306	5,464
MA1	BK_1653	Borsuka_C7_675	Mbuti.DG	-0.0671	0.015907	-4.22	1,429	1,635	28,207	-0.1382	0.034188	-4.042	255	337	5,305
MA1	BK_BB7_240	Borsuka_C7_675	Mbuti.DG	0.0671	0.016415	4.088	1,503	1,314	25,337	0.0541	0.035498	1.523	286	257	4,810
MA1	BK_CC7_335	Borsuka_C7_675	Mbuti.DG	0.0525	0.017391	3.018	1,377	1,240	23,560	0.0226	0.039075	0.579	252	241	4,429
MA1	PesteraMuierii	Borsuka_C7_675	Mbuti.DG	-0.0472	0.015493	-3.049	1,492	1,640	29,124	-0.041	0.035153	-1.166	288	313	5,469
BK_BB7_240	GoyetQ116	Borsuka_C7_675	Mbuti.DG	-0.107	0.017664	-6.06	1,126	1,396	22,841	-0.1246	0.040853	-3.049	197	253	4,212
BK_BB7_240	Vestonice16	Borsuka_C7_675	Mbuti.DG	-0.107	0.016374	-6.536	1,445	1,791	29,224	-0.1256	0.033392	-3.761	261	336	5,472
BK_BB7_240	Kostenki14	Borsuka_C7_675	Mbuti.DG	-0.0976	0.014301	-6.823	1,757	2,137	34,593	-0.1004	0.031756	-3.163	321	392	6,491
BK_BB7_240	LaBrana	Borsuka_C7_675	Mbuti.DG	-0.0913	0.015045	-6.066	1,728	2,075	33,506	-0.0743	0.032662	-2.274	320	371	6,292
BK_BB7_240	ElMiron	Borsuka_C7_675	Mbuti.DG	-0.0797	0.016429	-4.849	1,347	1,581	26,597	-0.0766	0.036354	-2.106	254	296	5,014
BK_BB7_240	Villabruna	Borsuka_C7_675	Mbuti.DG	-0.109	0.015181	-7.177	1,589	1,978	31,728	-0.1331	0.03285	-4.05	289	377	5,923
BK_BB7_240	Tianyuan	Borsuka_C7_675	Mbuti.DG	-0.0109	0.01537	-0.707	1,744	1,782	32,524	0.0031	0.033911	0.091	334	332	6,130
BK_BB7_240	Loschbour	Borsuka_C7_675	Mbuti.DG	-0.107	0.013741	-7.785	1,680	2,082	33,067	-0.0834	0.028899	-2.886	327	386	6,196
BK_BB7_240	Ust_Ishim	Borsuka_C7_675	Mbuti.DG	-0.0092	0.014605	-0.631	1,895	1,930	34,671	-0.0232	0.03164	-0.734	350	366	6,510
BK_BB7_240	Satsurblia	Borsuka_C7_675	Mbuti.DG	-0.032	0.017939	-1.785	1,098	1,171	20,095	-0.0528	0.038937	-1.357	204	227	3,779
BK_BB7_240	Sunghir3	Borsuka_C7_675	Mbuti.DG	-0.0933	0.01394	-6.69	1,803	2,174	34,632	-0.1244	0.031186	-3.988	326	419	6,505
BK_BB7_240	BK_1653	Borsuka_C7_675	Mbuti.DG	-0.1303	0.014383	-9.061	1,669	2,169	33,919	-0.1907	0.030545	-6.244	294	433	6,399
BK_BB7_240	MA1	Borsuka_C7_675	Mbuti.DG	-0.0671	0.016415	-4.088	1,314	1,503	25,337	-0.0541	0.035498	-1.523	257	286	4,810
BK_BB7_240	BK_CC7_335	Borsuka_C7_675	Mbuti.DG	-0.0031	0.016669	-0.187	1,491	1,500	28,657	-0.028	0.037588	-0.745	261	276	5,399
BK_BB7_240	PesteraMuierii	Borsuka_C7_675	Mbuti.DG	-0.1168	0.013873	-8.418	1,741	2,201	34,659	-0.1295	0.03046	-4.253	310	402	6,511
BK_CC7_335	GoyetQ116	Borsuka_C7_675	Mbuti.DG	-0.0938	0.018799	-4.99	1,088	1,313	21,315	-0.0638	0.041899	-1.523	196	222	3,927
BK_CC7_335	Vestonice16	Borsuka_C7_675	Mbuti.DG	-0.106	0.016444	-6.445	1,366	1,690	27,284	-0.1091	0.035853	-3.042	242	302	5,102
BK_CC7_335	Kostenki14	Borsuka_C7_675	Mbuti.DG	-0.0942	0.015581	-6.046	1,635	1,975	32,091	-0.0824	0.033831	-2.436	307	362	6,016
BK_CC7_335	LaBrana	Borsuka_C7_675	Mbuti.DG	-0.092	0.015356	-5.992	1,593	1,916	31,086	-0.0412	0.033307	-1.237	302	328	5,838
BK_CC7_335	ElMiron	Borsuka_C7_675	Mbuti.DG	-0.0639	0.01722	-3.708	1,293	1,470	24,842	-0.028	0.038188	-0.732	246	260	4,671
BK_CC7_335	Villabruna	Borsuka_C7_675	Mbuti.DG	-0.1022	0.015853	-6.448	1,491	1,831	29,621	-0.1111	0.033283	-3.338	263	328	5,512
BK_CC7_335	Tianyuan	Borsuka_C7_675	Mbuti.DG	-0.0099	0.015043	-0.657	1,626	1,658	30,324	0.0097	0.034508	0.281	294	289	5,700
BK_CC7_335	Loschbour	Borsuka_C7_675	Mbuti.DG	-0.0927	0.014186	-6.534	1,575	1,897	30,625	-0.0309	0.030893	-0.999	302	321	5,724
BK_CC7_335	Ust_Ishim	Borsuka_C7_675	Mbuti.DG	-0.0047	0.015257	-0.31	1,741	1,758	32,142	0.0134	0.034388	0.389	325	316	6,026
BK_CC7_335	Satsurblia	Borsuka_C7_675	Mbuti.DG	-0.0363	0.018857	-1.925	974	1,048	18,655	0.0166	0.044464	0.374	191	185	3,525
BK_CC7_335	Sunghir3	Borsuka_C7_675	Mbuti.DG	-0.0882	0.014375	-6.135	1,645	1,963	32,099	-0.1238	0.033923	-3.648	282	362	6,016
BK_CC7_335	BK_1653	Borsuka_C7_675	Mbuti.DG	-0.1214	0.015097	-8.043	1,594	2,035	31,484	-0.1657	0.033384	-4.963	287	401	5,934
BK_CC7_335	MA1	Borsuka_C7_675	Mbuti.DG	-0.0525	0.017391	-3.018	1,240	1,377	23,560	-0.0226	0.039075	-0.579	241	252	4,429
BK_CC7_335	BK_BB7_240	Borsuka_C7_675	Mbuti.DG	0.0031	0.016669	0.187	1,500	1,491	28,657	0.028	0.037588	0.745	276	261	5,399
BK_CC7_335	PesteraMuierii	Borsuka_C7_675	Mbuti.DG	-0.107	0.014715	-7.274	1,599	1,983	32,128	-0.0956	0.033796	-2.828	299	363	6,025
PesteraMuierii	GoyetQ116	Borsuka_C7_675	Mbuti.DG	0.0325	0.018234	1.782	1,363	1,277	25,099	0.026	0.039937	0.65	243	231	4,593
PesteraMuierii	Vestonice16	Borsuka_C7_675	Mbuti.DG	-0.009	0.015784	-0.571	1,657	1,688	32,500	-0.0225	0.034302	-0.656	295	309	6,045
PesteraMuierii	Kostenki14	Borsuka_C7_675	Mbuti.DG	0.0144	0.013453	1.07	2,138	2,077	39,851	0.0186	0.029497	0.63	402	387	7,425
PesteraMuierii	LaBrana	Borsuka_C7_675	Mbuti.DG	0.0145	0.013965	1.039	2,062	2,003	38,523	0.0559	0.03071	1.819	395	353	7,191
PesteraMuierii	ElMiron	Borsuka_C7_675	Mbuti.DG	0.0316	0.015727	2.008	1,566	1,470	29,331	0.0532	0.035072	1.515	312	280	5,483
PesteraMuierii	Villabruna	Borsuka_C7_675	Mbuti.DG	-0.0036	0.014208	-0.254	1,893	1,906	35,551	-0.0178	0.032415	-0.549	337	349	6,590
PesteraMuierii	Tianyuan	Borsuka_C7_675	Mbuti.DG	0.0989	0.013708	7.216	2,251	1,846	36,735	0.116	0.030746	3.772	436	346	6,870
PesteraMuierii	Loschbour	Borsuka_C7_675	Mbuti.DG	0.0083	0.012766	0.65	2,036	2,002	38,133	0.0453	0.027691	1.637	386	353	7,088
PesteraMuierii	Ust_Ishim	Borsuka_C7_675	Mbuti.DG	0.102	0.014016	7.279	2,439	1,988	40,085	0.0879	0.029821	2.949	455	382	7,479
PesteraMuierii	Satsurblia	Borsuka_C7_675	Mbuti.DG	0.0567	0.017798	3.187	1,340	1,196	23,247	0.099	0.038942	2.541	261	214	4,363
PesteraMuierii	Sunghir3	Borsuka_C7_675	Mbuti.DG	0.0145	0.013547	1.074	2,135	2,074	40,034	-0.0137	0.031931	-0.43	367	377	7,470
PesteraMuierii	BK_1653	Borsuka_C7_675	Mbuti.DG	-0.0223	0.013999	-1.59	1,920	2,008	38,680	-0.0879	0.032171	-2.731	319	381	7,244
PesteraMuierii	MA1	Borsuka_C7_675	Mbuti.DG	0.0472	0.015493	3.049	1,640	1,492	29,124	0.041	0.035153	1.166	313	288	5,469
PesteraMuierii	BK_BB7_240	Borsuka_C7_675	Mbuti.DG	0.1168	0.013873	8.418	2,201	1,741	34,659	0.1295	0.03046	4.253	402	310	6,511
PesteraMuierii	BK_CC7_335	Borsuka_C7_675	Mbuti.DG	0.107	0.014715	7.274	1,983	1,599	32,128	0.0956	0.033796	2.828	363	299	6,025

Supplementary Table 12 continued. *D*-statistics for *D*(W,X; Borsuka, Mbuti), related to STAR Methods (Ancient DNA: *Nuclear DNA analysis*).

Supplementary Figures

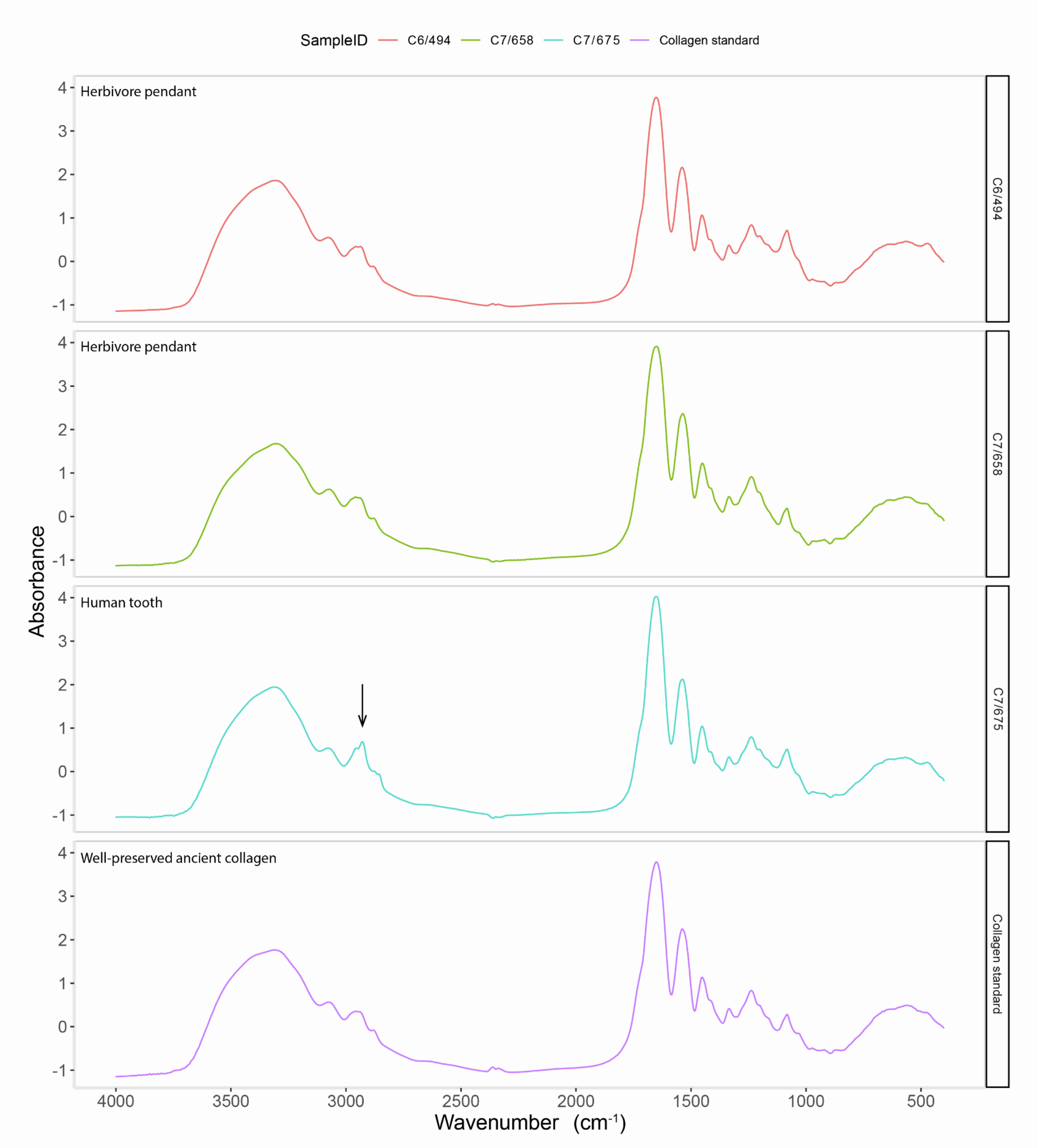

Supplementary Figure 1. FTIR spectra of Borsuka extracts compared with well-preserved collagen, related to STAR Methods (Collagen quality assessment). The higher intensity of the peak around 2900 cm^{-1} in the human extract is marked with an arrow.

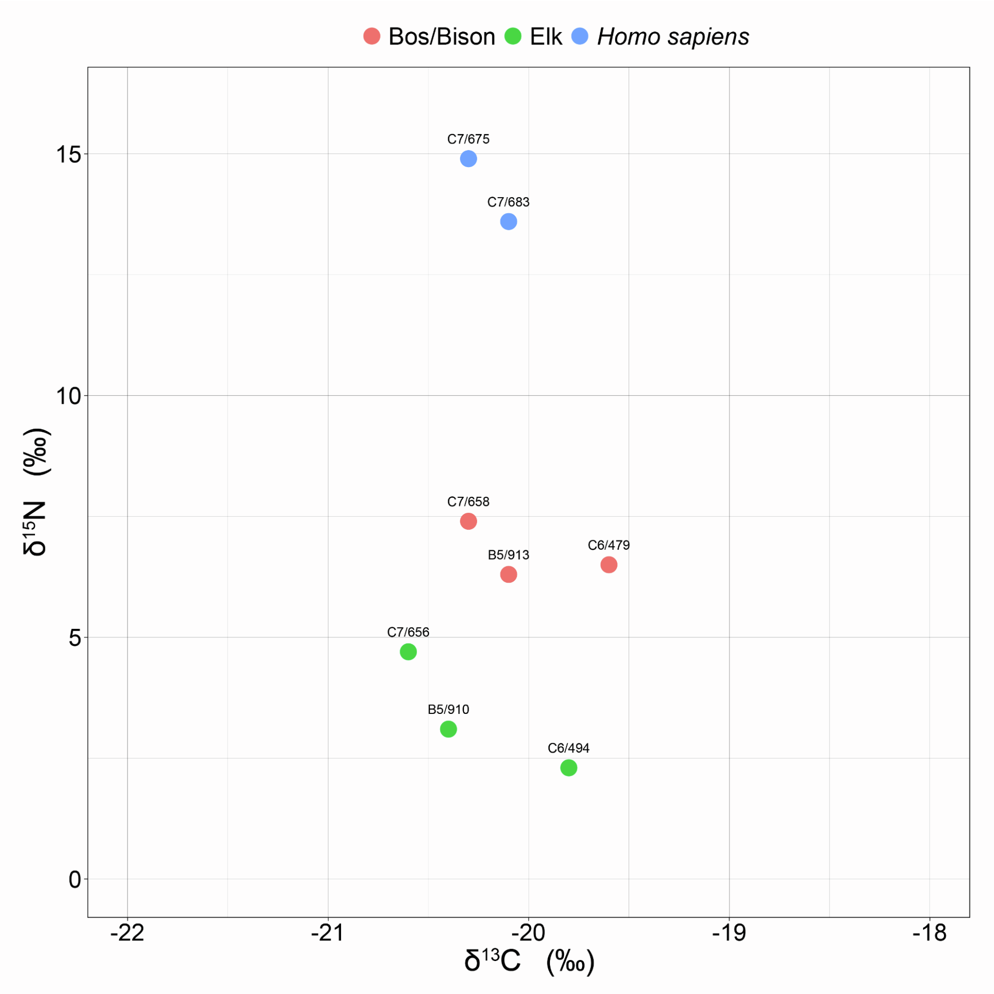

Supplementary Figure 2. Stable isotopic data of bulk collagen extracted from human teeth and perforated herbivore pendants from Layer VI at Borsuka Cave, related to STAR Methods (Collagen quality assessment).

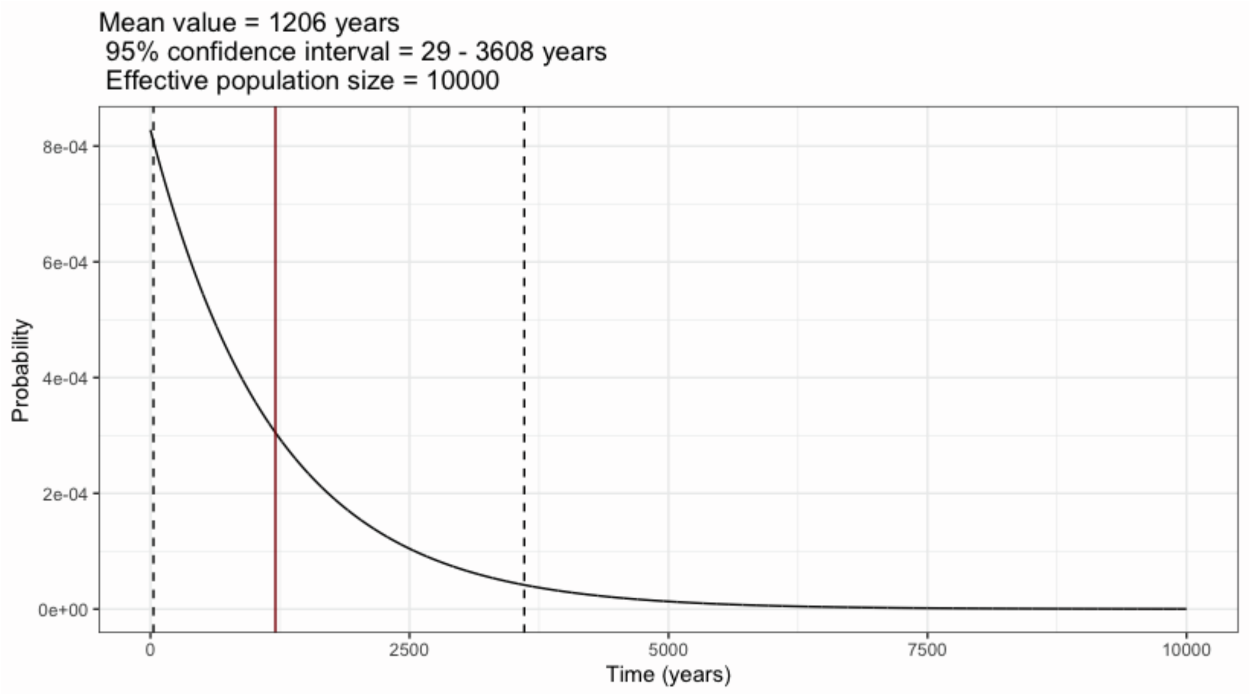

Supplementary Figure 3. The estimated TMRCA between two mtDNA genomes with no differences and a mutation rate of 2.67e-8 mutations per base pair per year across the full mtDNA genome, related to STAR Methods. The mean is shown in red and the 95% confidence intervals are represented by dashed lines.

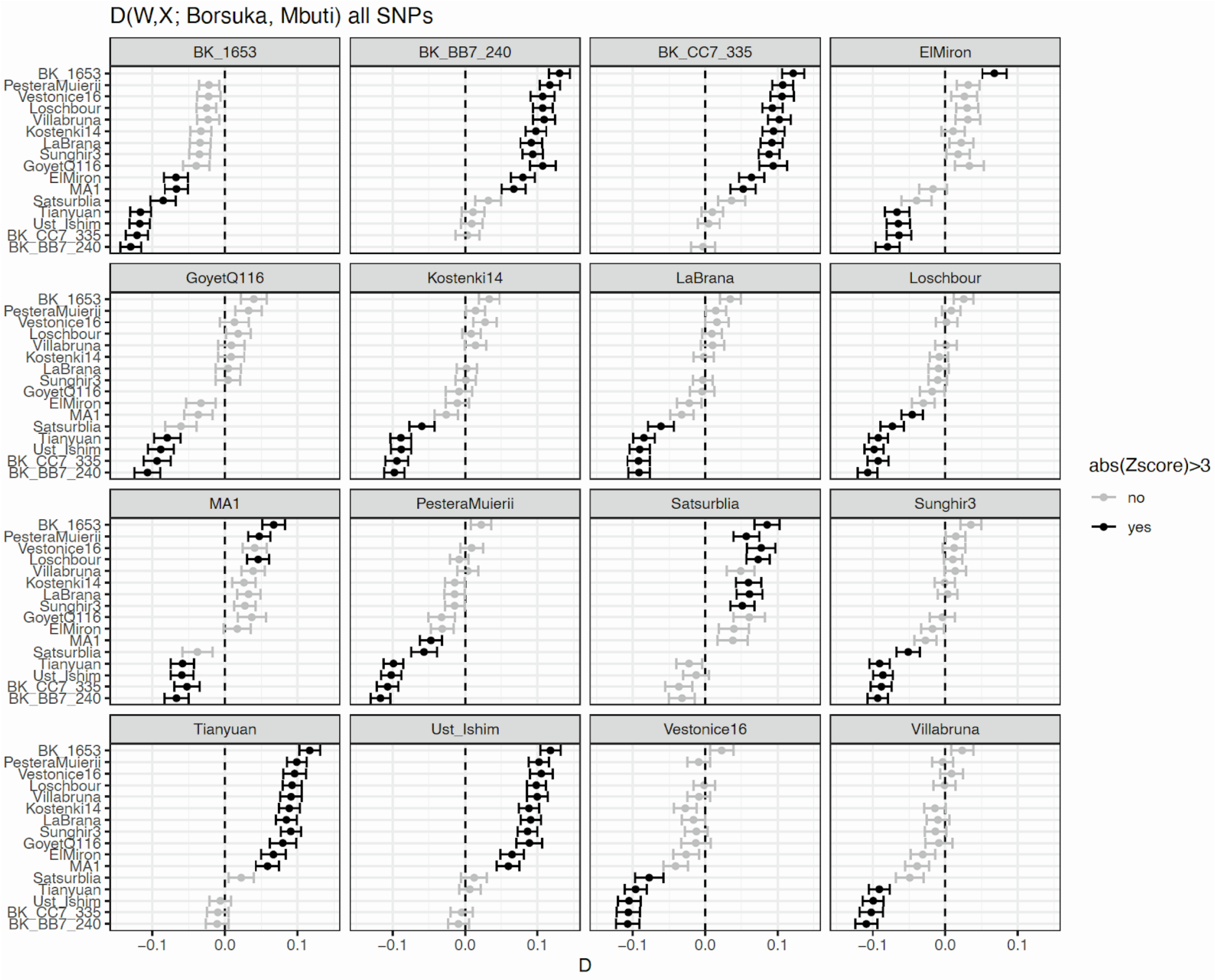

Supplementary Figure 4. D-statistics calculated with D(W,X; Borsuka, Mbuti) using between 14,423 and 40,085 overlapping SNPs via ADMIXTOOLs[S24] with the program *admixr*[S25], related to STAR Methods. W and X were a selection of ancient humans representing different genetic clusters of populations from the Upper Palaeolithic. Individuals in the W place are shown on the Y-axis and those in the X are placed as the header for each chart. Whiskers represent one standard error from the mean. See Supplementary Table 12 for values for each test.

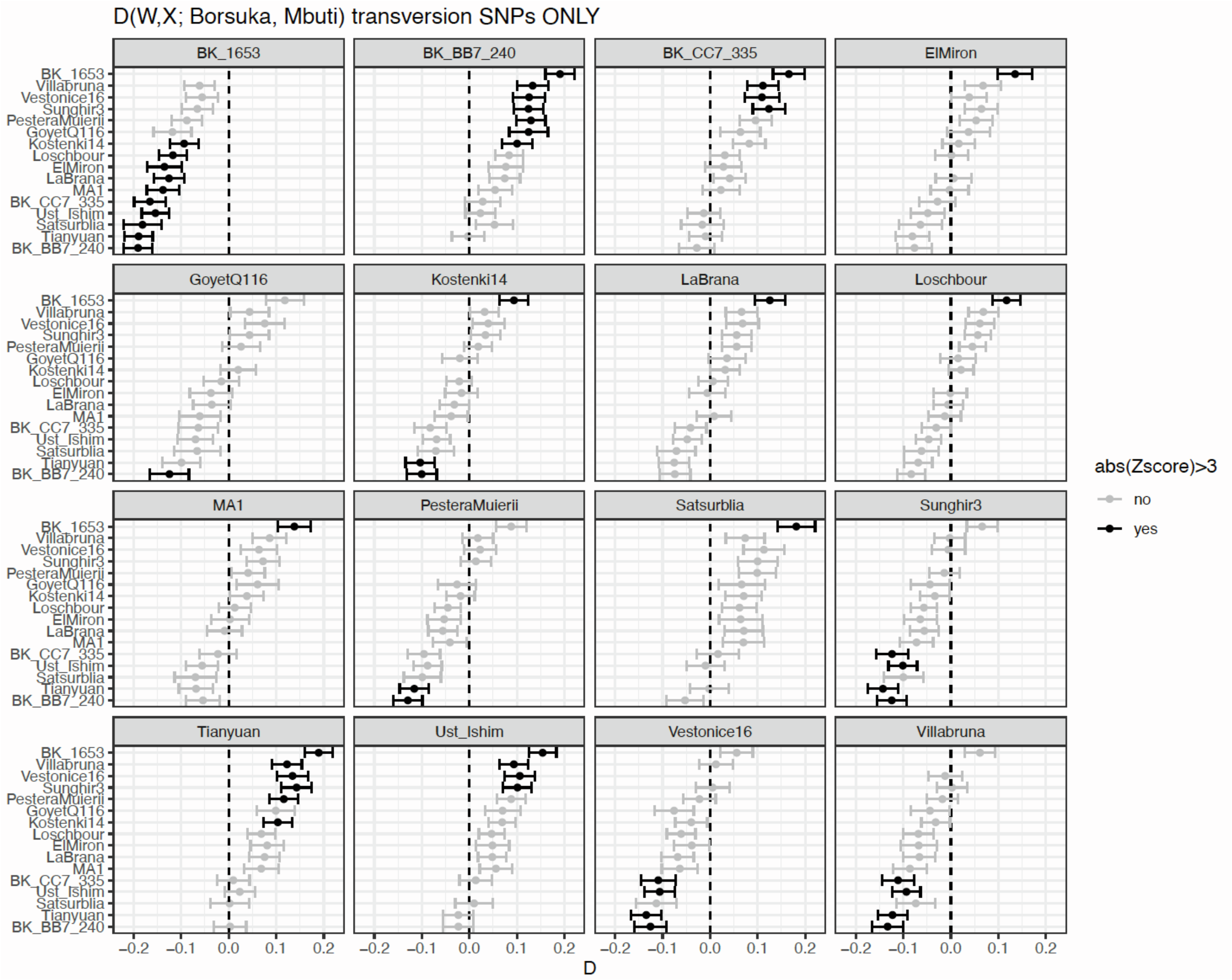

Supplementary Figure 5. D-statistics calculated with D(W,X; Borsuka, Mbuti) using between 2,643 and 7,479 overlapping transversion SNPs via ADMIXTOOLS[S24] with the program *admixr*[S25], related to STAR Methods. A selection of ancient humans representing different genetic clusters of populations from the Upper Palaeolithic were used in W and X. Individuals in the W place are shown on the Y-axis and those in the X are placed as the header for each chart. Whiskers represent one standard error from the mean. See Supplementary Table 12 for values for each test.

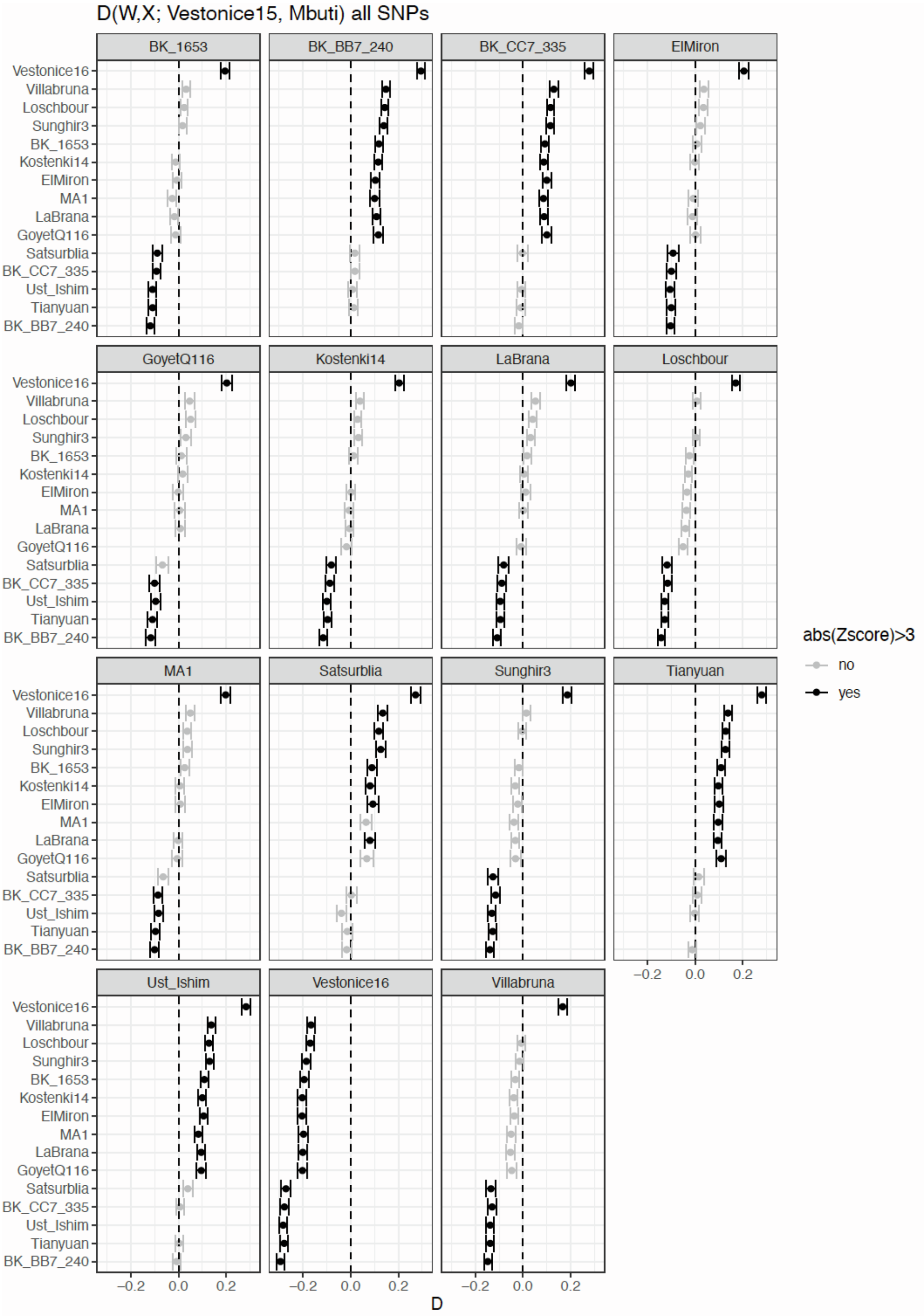

Supplementary Figure 6. D-statistics calculated with D(W,X; Vestonice15, Mbuti) using between 10,956 and 28,542 overlapping SNPs via ADMIXTOOLS[S24] with the program *admixr*[S25], related to STAR Methods. A selection of ancient humans representing different genetic clusters of populations from the Upper Palaeolithic were used in W and X. Individuals in the W place are shown on the Y-axis and those in the X are placed as the header for each chart. Whiskers represent one standard error from the mean.

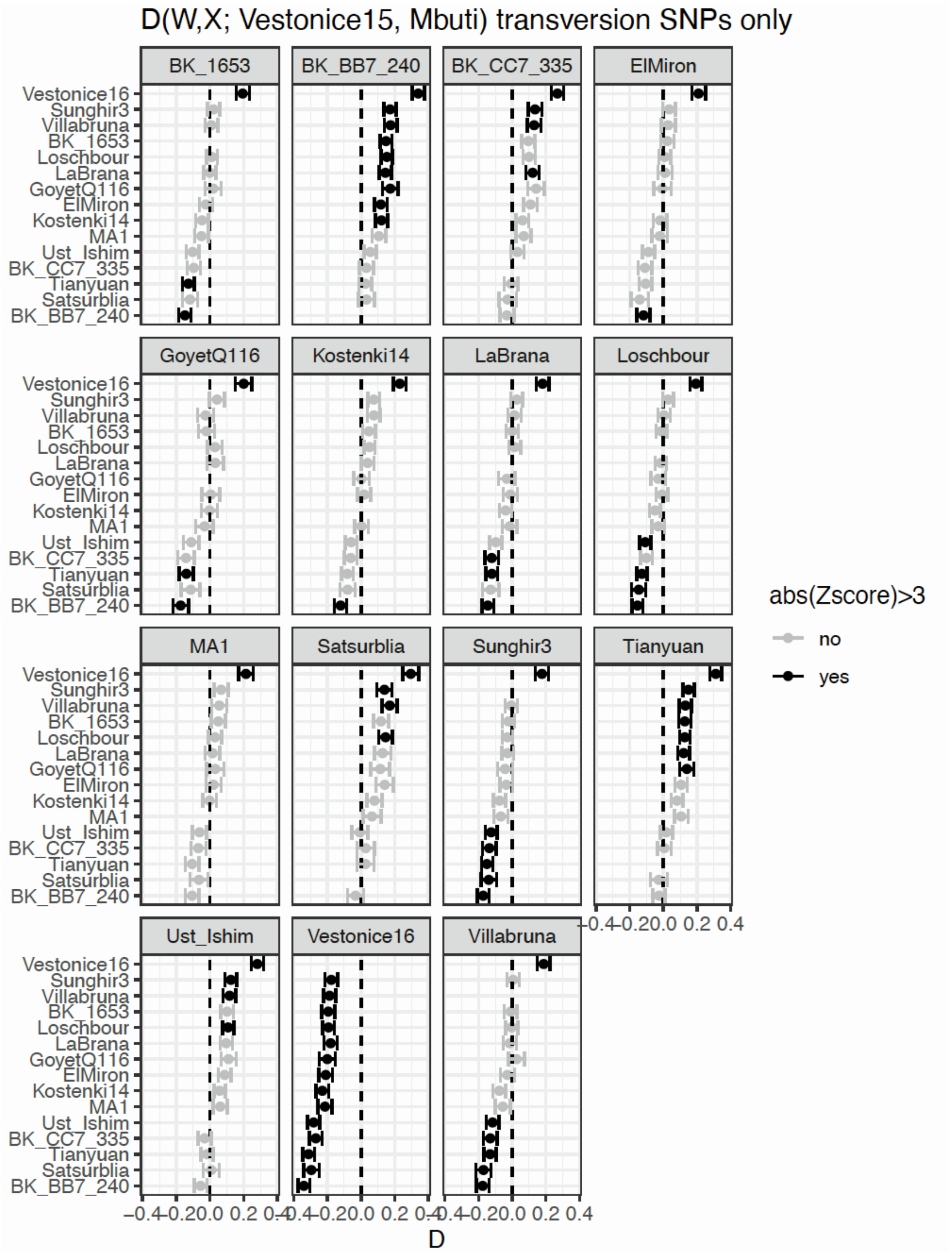

Supplementary Figure 7. D-statistics calculated with D(W,X; Vestonice15, Mbuti) using between 2,039 and 5,303 overlapping transversion SNPs via ADMIXTOOLS[S24] with the program *admixr*[S25], related to STAR Methods. A selection of ancient humans representing different genetic clusters of populations from the Upper Palaeolithic were used in W and X. Individuals in the W place are shown on the Y-axis and those in the X are placed as the header for each chart. Whiskers represent one standard error from the mean.

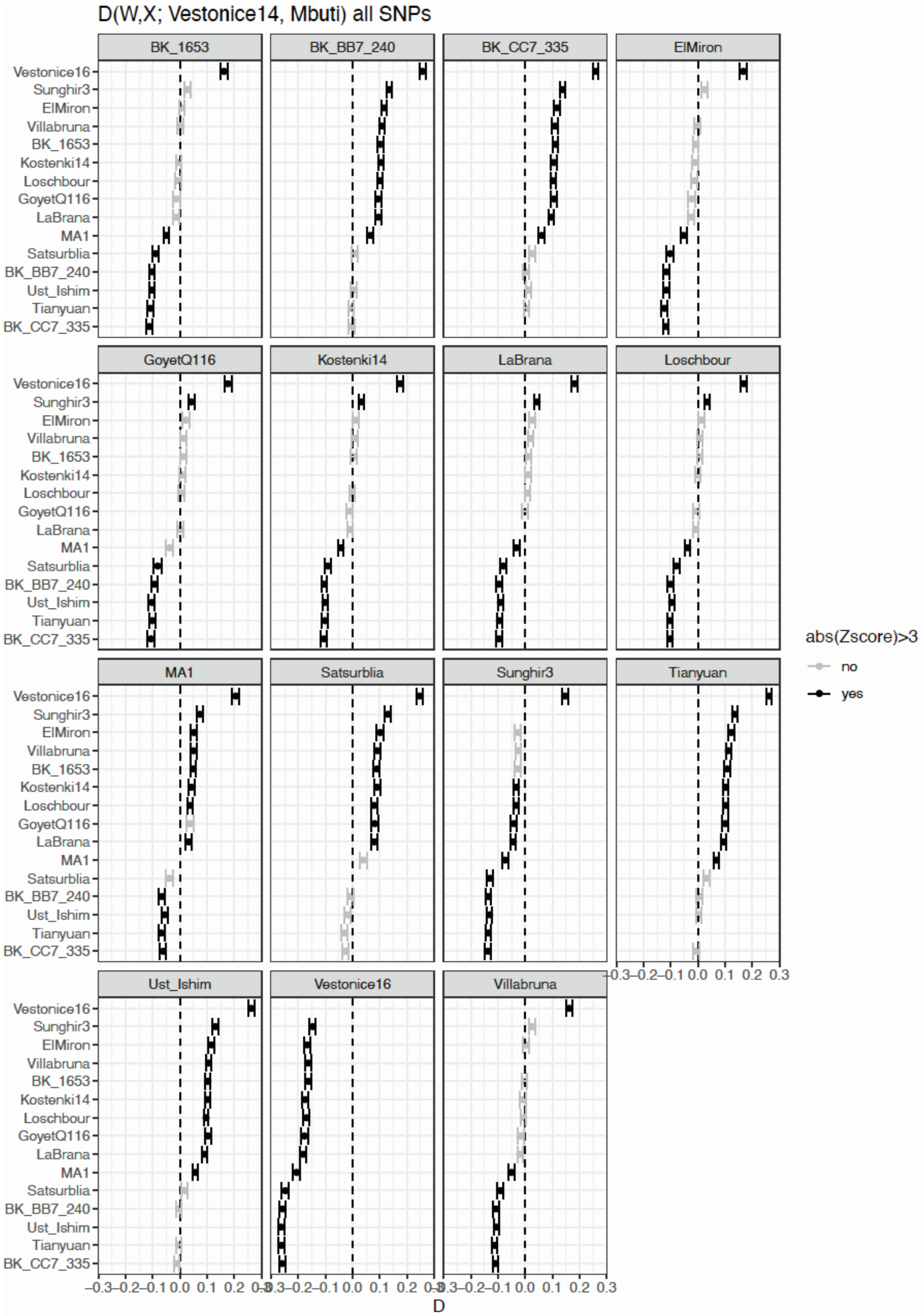

Supplementary Figure 8. D-statistics calculated with D(W,X; Vestonice14, Mbuti) using between 42,258 and 111,492 overlapping SNPs via ADMIXTOOLS[S24] with the program *admixr*[S25], related to STAR Methods. A selection of ancient humans representing different genetic clusters of populations from the Upper Palaeolithic were used in W and X. Individuals in the W place are shown on the Y-axis and those in the X are placed as the header for each chart. Whiskers represent one standard error from the mean.

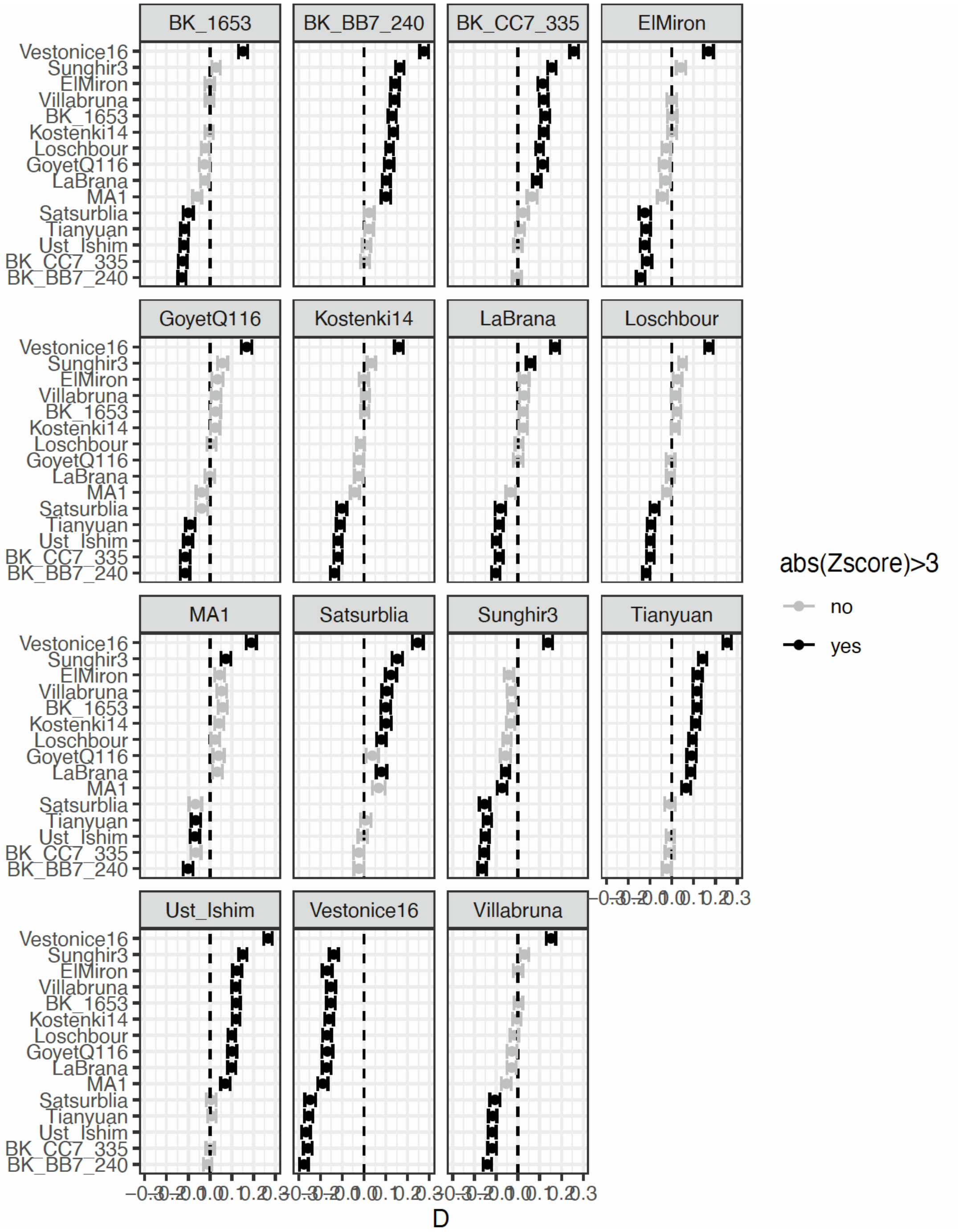

Supplementary Figure 9. D-statistics calculated with D(W,X; Vestonice14, Mbuti) using between 7,848 and 20,964 overlapping transversion SNPs via ADMIXTOOLS[S24] with the program *admixr*[S25], related to STAR Methods. A selection of ancient humans representing different genetic clusters of populations from the Upper Palaeolithic were used in W and X. Individuals in the W place are shown on the Y-axis and those in the X are placed as the header for each chart. Whiskers represent one standard error from the mean.

Supplementary References

S1. Bronk-Ramsey, C. (2009). Bayesian Analysis of Radiocarbon Dates. Radiocarbon *51*, 337–360.

S2. Reimer, P.J., Austin, W.E.N., Bard, E., Bayliss, A., Blackwell, P.G., Bronk-Ramsey, C., Butzin, M., Cheng, H., Lawrence Edwards, R., Friedrich, M., et al. (2020). The IntCal20 Northern Hemisphere Radiocarbon Age Calibration Curve (0–55 cal kBP). Radiocarbon *62*, 725–757.

S3. Fu, Q., Li, H., Moorjani, P., Jay, F., Slepchenko, S.M., Bondarev, A.A., Johnson, P.L.F., Aximu-Petri, A., Prüfer, K., de Filippo, C., et al. (2014). Genome sequence of a 45,000-year-old modern human from western Siberia. Nature *514*, 445–449.

S4. Fu, Q., Mittnik, A., Johnson, P.L.F., Bos, K., Lari, M., Bollongino, R., Sun, C., Giemsch, L., Schmitz, R., Burger, J., et al. (2013a). A revised timescale for human evolution based on ancient mitochondrial genomes. Curr. Biol. *23*, 553–559.

S5. Higham, T.F.G., Bronk Ramsey, C., Brock, F., Baker, D., and Ditchfield, P. (2007). Radiocarbon dates from the oxford AMS system: Archaeometry datelist 32. Archaeometry *49*, S1–S60.

S6. Fu, Q., Meyer, M., Gao, X., Stenzel, U., Burbano, H.A., Kelso, J., and Pääbo, S. (2013b). DNA analysis of an early modern human from Tianyuan Cave, China. Proc. Natl. Acad. Sci. U. S. A. *110*, 2223–2227.

S7. Marom, A., McCullagh, J.S.O., Higham, T.F.G., Sinitsyn, A.A., and Hedges, R.E.M. (2012). Single amino acid radiocarbon dating of Upper Paleolithic modern humans. Proc. Natl. Acad. Sci. U. S. A. *109*, 6878–6881.

S8. Krause, J., Briggs, A.W., Kircher, M., Maricic, T., Zwyns, N., Derevianko, A., and Pääbo, S. (2010). A complete mtDNA genome of an early modern human from Kostenki, Russia. Curr. Biol. *20*, 231–236.

S9. Kutschera, W. 4.4 Radiocarbon dating of the Iceman Ötzi with accelerator mass spectrometry. https://www2.chemistry.msu.edu/courses/CEM485/Lectures/IcemanAge.pdf.

S10. Ermini, L., Olivieri, C., Rizzi, E., Corti, G., Bonnal, R., Soares, P., Luciani, S., Marota, I., De Bellis, G., Richards, M.B., et al. (2008). Complete mitochondrial genome sequence of the Tyrolean Iceman. Curr. Biol. *18*, 1687–1693.

S11. Rasmussen, M., Li, Y., Lindgreen, S., Pedersen, J.S., Albrechtsen, A., Moltke, I., Metspalu, M., Metspalu, E., Kivisild, T., Gupta, R., et al. (2010). Ancient human genome sequence of an extinct Palaeo-Eskimo. Nature *463*, 757–762.

S12. Gilbert, M.T.P., Kivisild, T., Grønnow, B., Andersen, P.K., Metspalu, E., Reidla, M., Tamm, E., Axelsson, E., Götherström, A., Campos, P.F., et al. (2008). Paleo-Eskimo mtDNA genome reveals matrilineal discontinuity in Greenland. Science *320*, 1787–1789.

S13. Hedges, R.E.M., Housley, R.A., Bronk-Ramsey, C., and van Klinken, G.J. (1992). Radiocarbon dates from the oxford AMS system: Archaeometry datelist 15. Archaeometry *34*, 337–357.

S14. Fewlass, H., Talamo, S., Kromer, B., Bard, E., Tuna, T., Fagault, Y., Sponheimer, M., Ryder, C., Hublin, J.-J., Perri, A., et al. (2019). Direct radiocarbon dates of mid Upper Palaeolithic human remains from Dolní Věstonice II and Pavlov I, Czech Republic. Journal of Archaeological Science: Reports *27*, 102000.

S15. Fewlass, H., Talamo, S., Wacker, L., Kromer, B., Tuna, T., Fagault, Y., Bard, E., McPherron, S.P., Aldeias, V., Maria, R., et al. (2020). A 14C chronology for the Middle to Upper Palaeolithic transition at Bacho Kiro Cave, Bulgaria. Nature Ecology & Evolution *4*, 794–801.

S16. Hublin, J.-J., Sirakov, N., Aldeias, V., Bailey, S., Bard, E., Delvigne, V., Endarova, E., Fagault, Y., Fewlass, H., Hajdinjak, M., et al. (2020). Initial Upper Palaeolithic Homo sapiens from Bacho Kiro Cave, Bulgaria. Nature *581*, 299–302.

S17. Trinkaus, E., Moldovan, O., Milota, S., Bîlgăr, A., Sarcina, L., Athreya, S., Bailey, S.E., Rodrigo, R., Mircea, G., Higham, T., et al. (2003). An early modern human from the Peştera cu Oase, Romania. Proc. Natl. Acad. Sci. U. S. A. *100*, 11231–11236.

S18. Fu, Q., Hajdinjak, M., Moldovan, O.T., Constantin, S., Mallick, S., Skoglund, P., Patterson, N., Rohland, N., Lazaridis, I., Nickel, B., et al. (2015). An early modern human from Romania with a recent Neanderthal ancestor. Nature *524*, 216–219.

S19. Soficaru, A., Doboş, A., and Trinkaus, E. (2006). Early modern humans from the Peştera Muierii, Baia de Fier, Romania. Proc. Natl. Acad. Sci. U. S. A. *103*, 17196–17201.

S20. Fu, Q., Posth, C., Hajdinjak, M., Petr, M., Mallick, S., Fernandes, D., Furtwängler, A., Haak, W., Meyer, M., Mittnik, A., et al. (2016). The genetic history of Ice Age Europe. Nature *534*, 200–205.S21. Benazzi, S., Slon, V., Talamo, S., Negrino, F., Peresani, M., Bailey, S.E., Sawyer, S., Panetta, D., Vicino, G., Starnini, E., et al. (2015). Archaeology. The makers of the Protoaurignacian and implications for Neandertal extinction. Science *348*, 793–796.

S22. Devièse, T., Karavanić, I., Comeskey, D., Kubiak, C., Korlević, P., Hajdinjak, M., Radović, S., Procopio, N., Buckley, M., Pääbo, S., et al. (2017). Direct dating of Neanderthal remains from the site of Vindija Cave and implications for the Middle to Upper Paleolithic transition. Proc. Natl. Acad. Sci. U. S. A. *114*, 10606–10611.

S23. Prüfer, K., de Filippo, C., Grote, S., Mafessoni, F., Korlević, P., Hajdinjak, M., Vernot, B., Skov, L., Hsieh, P., Peyrégne, S., et al. (2017). A high-coverage Neandertal genome from Vindija Cave in Croatia. Science *358*, 655–658.

S24. Patterson, N., Moorjani, P., Luo, Y., Mallick, S., Rohland, N., Zhan, Y., Genschoreck, T., Webster, T., and Reich, D. (2012). Ancient admixture in human history. Genetics *192*, 1065–1093.

S25. Petr, M., Vernot, B., and Kelso, J. (2019). admixr—R package for reproducible analyses using ADMIXTOOLS. Bioinformatics *35*, 3194–3195.
